# Modern antidiabetic therapy by sodium-glucose cotransporter 2 inhibitors, glucagon-like peptide 1 receptor agonists, and dipeptidyl peptidase 4 inhibitors against cardiovascular diseases

**DOI:** 10.1016/j.pharmr.2025.100082

**Published:** 2025-07-10

**Authors:** Sebastian Steven, Marin Kuntic, Thomas Münzel, Andreas Daiber

**Affiliations:** 1 Department for Cardiology 1, University Medical Center of the Johannes Gutenberg-University, Mainz, Germany; 2 Center for Thrombosis and Hemostasis, University Medical Center of the Johannes Gutenberg-University, Mainz, Germany; 3 German Center for Cardiovascular Research (DZHK), Partnersite Rhine-Main, Mainz, Germany; 4 Department of Cardiology, Goethe University Frankfurt, Germany

## Abstract

Diabetes and related metabolic diseases have a high prevalence with increasing incidence and create a significant socioeconomic burden by their contribution to global mortality and disability-adjusted life years. According to data from the Global Burden of Disease Study, high fasting plasma glucose and high total cholesterol rank third and fourth in the list of global health risk factors, just behind high blood pressure and smoking. Diabetes adversely affects endothelial and cardiac function, thereby contributing significantly to the development and progression of cardiovascular diseases, which represents the leading health risk factors and causes of death worldwide. Oxidative stress and inflammation play a key role in the pathophysiology underlying diabetes mellitus and the associated cardiometabolic complications, such as metabolic dysfunction-associated fatty liver disease, hypertension, atherosclerosis, myocardial ischemia/reperfusion, and heart failure. Here, we highlight the beneficial effects of the modern antidiabetic drug classes of dipeptidyl peptidase 4 inhibitors, glucagon-like peptide 1 receptor agonists, and sodium-glucose cotransporter 2 inhibitors on overall and cardiovascular mortality of diabetic individuals, with particular emphasis on their effects on oxidative stress, inflammation, and endothelial dysfunction. We discuss the mechanisms of action and pleiotropic beneficial effects and compare them with standard diabetic and cardiovascular therapy.

**Significance Statement:**

Modern antidiabetic drugs confer organ protection that goes beyond simple glucose-lowering. SGLT2 inhibitors and incretin-based drugs possess direct reno-, vasculo-, and cardioprotective effects that are based on potent antioxidant and anti-inflammatory properties. Other pleiotropic effects comprise improved lipid handling and weight loss, prevention of thrombosis and ischemic heart damage, and beneficial regulation of nitric oxide signaling and epigenetic and microbiotic pathways.

## I Introduction

### A Historical background

The term diabetes mellitus (DM) has Greek and Latin roots and means “honey-sweet flow-through,” which is related to the higher glucose concentration in the urine of diabetic individuals. Medical scribes of the ancient world had diagnosed DM by the sweet taste of the urine of diabetic individuals (the earliest written description on the *Ebers Papyrus* dates back to around 552 BC).^1^ The first detailed description of DM was documented by Thomas Willis in 1675. Before the first medications, individuals with diabetes had a short life expectancy, and the only way to minimize diabetic complications was adherence to a low-carbohydrate diet. The disease is characterized by abnormal glucose uptake into cells and altered metabolism due to the lack of insulin or reduced insulin signaling, leading to impaired energy metabolism and high levels of toxic metabolites such as ketones and aldehydes.^2^ Diabetes mellitus can be separated into 2 major forms. Type 1 DM (T1DM) is characterized by the complete loss of insulin production, mostly due to autoimmune-dependent elimination of the islet cells; surgical removal of the pancreas in cases of pancreatic cancer can also lead to insulin dependence. T1DM is easier to recognize in children or young adults^3^ and was therefore previously called juvenile diabetes. In contrast, type 2 DM (T2DM) is characterized by impaired insulin production and/or receptor signaling and magnified by the obesity epidemic and an unhealthy lifestyle, which disturbs insulin signaling via not only the production of unhealthy mediators from adipose tissue but also the exaggerated fueling of glucose-dependent pathways. Although T2DM was previously observed more frequently in the elderly, it is also now detected in children and young and middle-aged individuals due to overnutrition and a sedentary lifestyle. Diabetes mellitus is diagnosed by fasting blood glucose levels of ≥126 mg/dL (≥7.0 mmol/L) or HbA1c values of >7.5% for moderate hyperglycemia (>9.0% for severe DM), which represents glycated hemoglobin concentrations and provides the glycemic status average of the last 7–8 weeks (also shown in Table 1 according to the actual American Diabetes Association [ADA] guidelines).^4^

**Table 1:** Criteria for the diagnosis of diabetes in nonpregnant individuals Reproduced from American Diabetes Association Professional Practice Committee^4^ with permission.

A1C ≥6.5% (≥48 mmol/mol). The test should be performed in a laboratory using a method that is NGSP certified and standardized to the DCCT assay.^a^
OR
FPG ≥126 mg/dL (≥7.0 mmol/L). Fasting is defined as no caloric intake for at least 8 h.^a^
OR
2-h PG ≥200 mg/dL (≥11.1 mmol/L) during OGTT. The test should be performed as described by the WHO, using a glucose load containing the equivalent of 75 g anhydrous glucose dissolved in water.^a^
OR
In an individual with classic symptoms of hyperglycemia or hyperglycemic crisis, a random plasma glucose ≥200 mg/dL (≥11.1 mmol/L). Random is any time of the day without regard to time since previous meal.

2-h PG, 2-hour plasma glucose; DCCT, Diabetes Control and Complications Trial; FPG, fasting plasma glucose; NGSP, National Glycohemoglobin Standardization Program; OGTT, oral glucose tolerance test; WHO, World Health Organization.

a In the absence of unequivocal hyperglycemia, diagnosis requires 2 abnormal test results obtained at the same time (eg, A1C and FPG) or at 2 different time points.

### B Contribution of diabetes and metabolic syndrome to the Global Burden of Disease

**Fig. 1:**
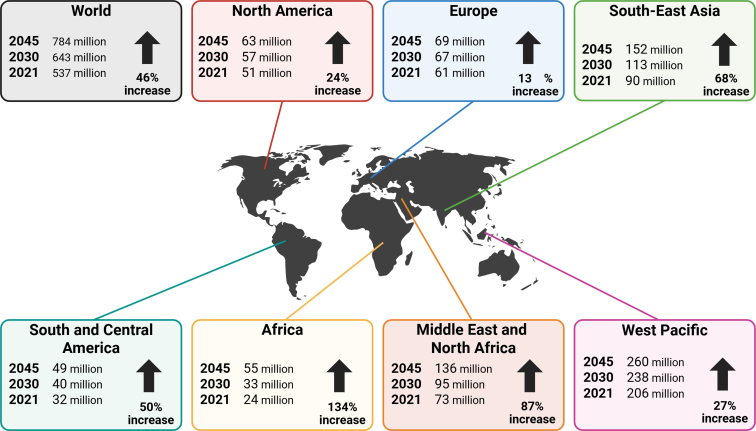
Prevalence of diabetes. Number of people with diabetes worldwide and per IDF Region in 2021–2045 (20–79 years). Adapted from the IDF Diabetes Atlas^6^ with permission, licensed under a Creative Commons Attribution-NonCommercial-NoDerivatives 4.0 International License.

The prevalence of DM worldwide was 422 million people in the year 2014, which means 8.5% of the adult population, and represents an almost 4-fold increase compared with the year 1980.^5^ An up-to-date picture of the prevalence of diabetes is shown in Fig. 1.^6^ Within the last 20 years, the GBD and mortality has shifted from communicable childhood diseases toward noncommunicable diseases such as atherosclerotic and metabolic diseases including T2DM.^7^^,^^8^ Diabetes mellitus is associated with 1.5 million global deaths per year as a direct consequence of glucotoxicity, whereas approximately 2.2 million deaths are caused by indirect effects of hyperglycemia, for example, increased risk of cardiovascular (CV) events such as myocardial infarction (MI) or stroke.^5^ Accordingly, cardiovascular disease (CVD) is common in diabetic individuals, illustrated by a meta-analysis reporting a hazard ratio (HR) of 3.42 (95% confidence interval [CI], 2.23–5.23) for CV mortality in diabetic individuals.^9^ Another study found that 1% higher HbA1c levels resulted in a higher HR for primary composite outcomes such as heart failure hospitalization, MI, CV death, hospitalization for worsening heart failure, and death from all causes, with a range of 1.22–1.25 for each outcome.^10^ In general, comorbidities such as obesity, diabetes, and metabolic syndrome can significantly impact clinical outcomes, for example, the development of ischemic heart disease and other CVDs.^11^ A large-scale national study with more than 55 million participants found that hyperlipidemia and hypertension had the strongest associations with MI (odds ratio [OR], 8.39 and 3.11), followed by diabetes and metabolic dysfunction-associated fatty liver disease (MAFLD; OR, 1.89 and 1.5).^12^ Other risk factors associated with MI included smoking, age above 65 years, and male sex (OR, 2.83, 1.47, and 1.53). People with diabetes also exhibit suboptimal responses to some cardioprotective therapies.^13^ These findings highlight the importance of understanding the association between comorbidities and CVD risk in patients with established CVDs.

**Fig. 2:**
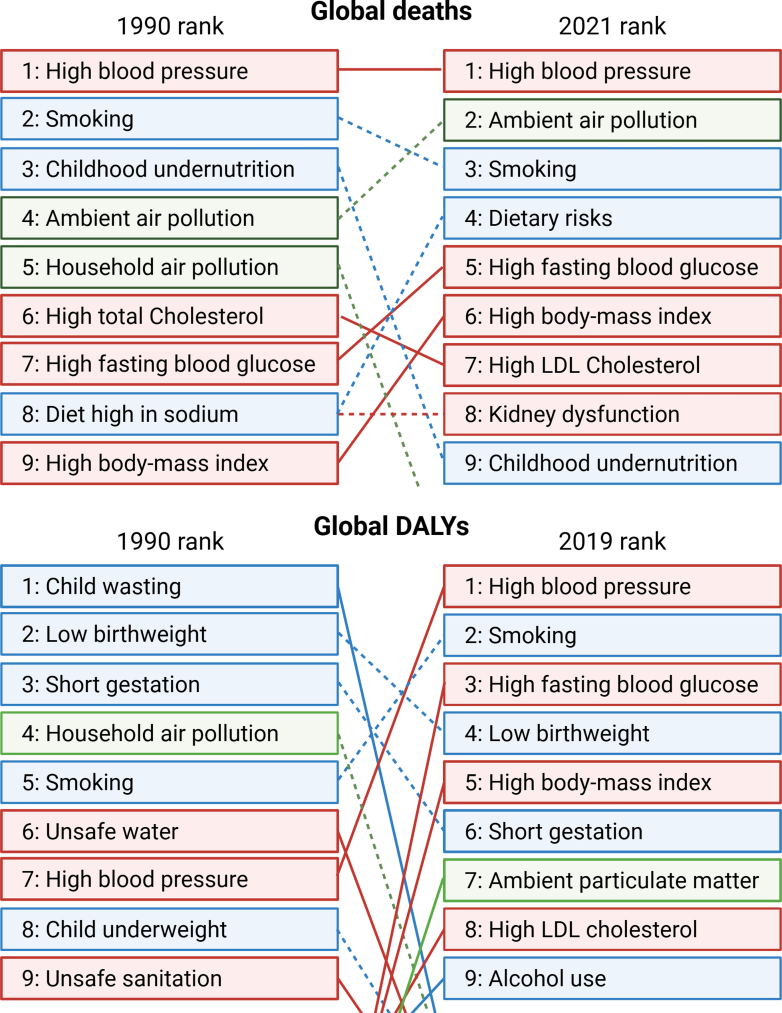
Global health risk factors for deaths and disability-adjusted life years (DALYs). The top 9 risk factors contributing to global deaths are shown for the year 1990 compared to 2021. Red color, metabolic risks; blue color, behavioural risks; green color, environmental or occupational risks. Based on data on deaths of the Global Burden of Disease (GBD) Study. Order for the year 2021 was adapted from report in a previous work.^15^ Data on DALYs for the year 1990 compared to 2019 from GBD study in Collaborators GBDRF^16^ was reused with permission. This article is available under the terms of the Creative Commons Attribution License (CC BY).

In the United States, the economic burden imposed by DM is enormous, as envisaged by almost 240 billion USD in national health care costs plus a loss of approximately 90 billion USD due to lower productivity, unemployment, and years of life lost in the year 2017.^14^ These numbers are in agreement with the more recent Global Burden of Disease (GBD) data, indicating that the importance of diabetes and other metabolic risk factors increased over the last decades, which is also reflected by ranking DM (high fasting blood glucose) fifth among the global health risk factors for premature death in the year 2021 compared with the seventh position in 1990 (Fig. 2).^15^^,^^16^ High fasting blood glucose was also ranked third among the global health risk factors for chronic diseases (reflected by disability-adjusted life years) in the year 2019. Growing importance for the GBD was also reported for other metabolic risk factors such as hyperlipidemia (high total cholesterol) and obesity (high body mass index) that are now ranked seventh and fourth, respectively, mainly reflecting the impact of Western lifestyle (eg, unhealthy diet, physical inactivity, and probably environmental triggers).

### C Standard glucose-lowering therapy and cardiovascular complications

Decreasing glucose levels reduces the risk of microvascular complications in patients with T2DM. A drop in glycated hemoglobin (HbA1c) by 1% is linked to improved long-term outcomes.^17^ Intensive glucose-lowering therapy has been shown to improve macrovascular outcomes in T2DM patients.^18^ The ADA and European Association for the Study of Diabetes recommend starting with weight reduction, exercise, dietary changes, and lifestyle modifications before medical therapy.^19^ Metformin is often used as an initial therapy for T2DM treatment due to its glycemic efficacy, absence of weight gain, and favorable cost.

**Fig. 3:**
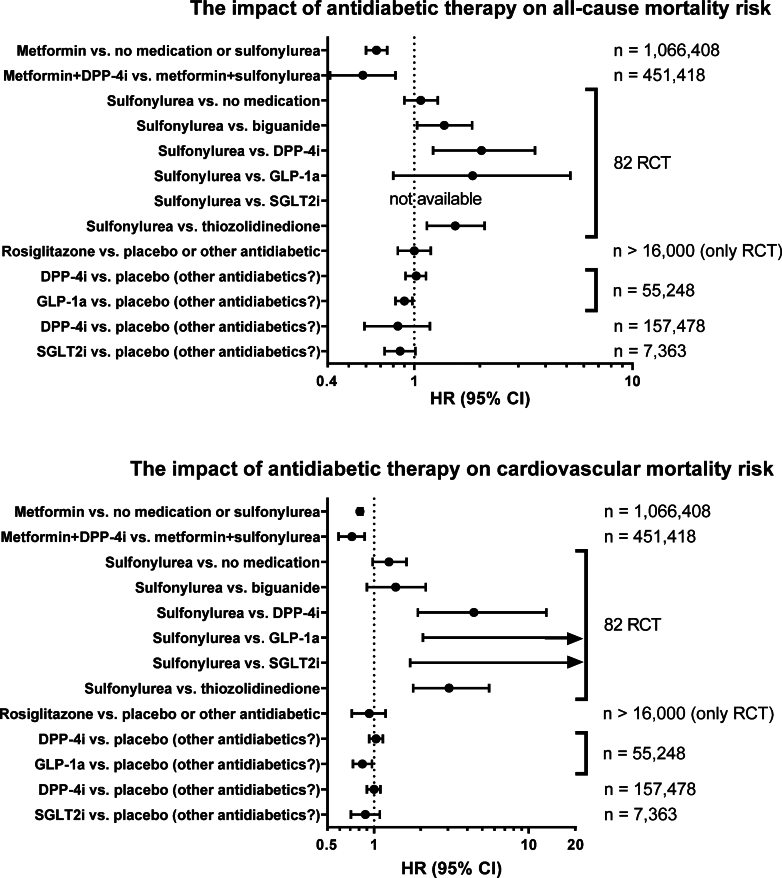
Different antidiabetic therapies reduce the HR for all-cause or cardiovascular mortality in T2DM patients. Of note, the control group was heterogeneous as most T2DM patients had basic antidiabetic therapy (some participants had no medication at all or sulfonylurea treatment). Data were taken from Han et al^24^ for metformin versus no medication or sulfonylurea, from Wang et al^25^ for metformin+dipeptidyl peptidase 4 inhibitors (DPP-4i) versus metformin+sulfonylurea, from Bain et al^26^ for sulfonylurea versus other medications, from Cheng et al^27^ for rosiglitazone versus no medication or other antidiabetics, from Zhang et al^28^ for DPP-4i or glucagon-like peptide 1 analogs (GLP-1a) versus placebo (or other antidiabetics), from Liu et al^29^ versus DPP-4i versus no DPP-4i and from Toyama et al^30^ for SGLT2i versus no SGLT2i. RCT, randomized controlled trials. An in-depth overview of cardioprotective profile of GLP-1RA is shown in Table 2 and of SGLT2i in Figure 5.

A historic breakthrough in therapy for T1DM was the discovery of insulin in 1922, which led to the awarding of the Nobel Prize in Physiology or Medicine to Banting and Macleod in 1923. Significant support in their discovery of insulin came from the researchers Best and Collip. Shortly after, Eli Lilly and then Nordisk Insulin Laboratory started to isolate insulin from animal pancreas, purified and distributed it to diabetic patients.^1^ Genentech expressed the first recombinant DNA of human insulin in *Escherichia coli* in 1978, and a commercialization agreement was signed with Lilly. In 1982, Humulin was introduced in the market, the first human recombinant insulin based on rDNA technology.^1^ As for patients with T1DM, the first-line therapy is intensive insulin therapy.^19^ Here, we focus our review on the therapy of T2DM. Glycemic control using medications (lowering the glycated hemoglobin [HbA1c] by only 1%) improves long-term outcomes in patients with T2DM, associated with a reduction of microvascular damage^17^ and beneficial effects on macrovascular function,^18^ respectively. According to the guidelines of the ADA 2025 and the European Association for the Study of Diabetes 2023, nonpharmacological treatment strategies (eg, weight reduction, exercise, dietary measures, and intensive lifestyle modification) represent the first-line therapy for newly diagnosed T2DM. If nonpharmacological measures fail, therapy of T2DM starts with the biguanide metformin due to its glycemic efficacy, absence of weight gain and hypoglycemia, general tolerability, and favorable cost. Mouse data suggest that a large part of the beneficial actions of metformin is based on activation of the AMP-activated protein kinase (AMPK),^20^ a central regulator of energy metabolism and antioxidant as well as anti-inflammatory defense.^21^^,^^22^ Other glucose-lowering agents include sulfonylureas (eg, glibenclamide and modulators of potassium channels), meglitinides (glinides and modulators of K_ATP_-channels), and peroxisome proliferator-activated receptors agonists (glitazones), as well as the *α*-glucosidase inhibitors, saccharides that act as competitive inhibitors of enzymes that digest carbohydrates after meal uptake in the intestine.^23^ A comparison of the efficacy of different therapies is presented in Fig. 3.^24^, ^25^, ^26^, ^27^, ^28^, ^29^, ^30^

## II Cardiovascular pathophysiology of diabetes and metabolic syndrome

### A Major cardiovascular complications of diabetes and metabolic syndrome

**Fig. 4:**
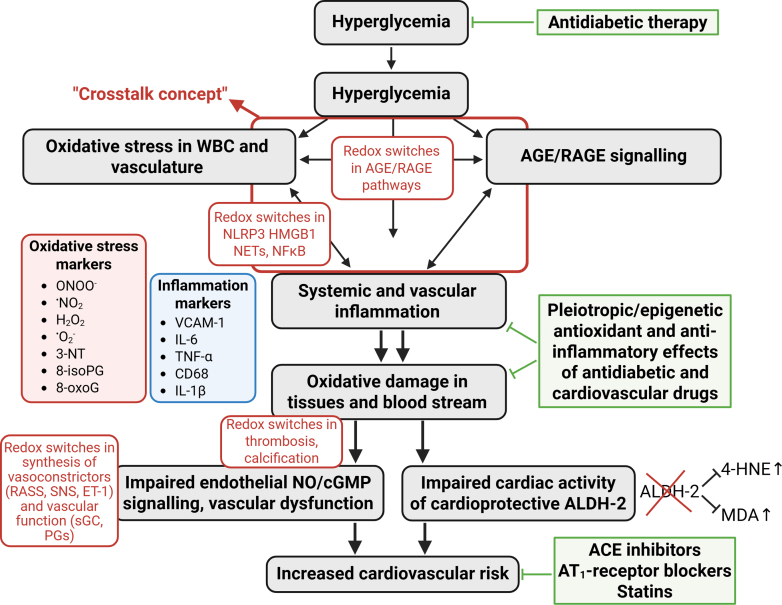
Cardiovascular pathophysiology of diabetes. Extension of the crosstalk concept from ROS sources to pathways initiated by hyperglycemia and glucotoxicity (fructose and sorbitol overproduction) such as inflammation (typical diabetic markers in the blue elliptic box), AGE/RAGE signaling (typical AGE members: precursor methylglyoxal and protein adduct N^*ε*^-(carboxylethyl)-l-lysine), synthesis of vasoconstrictors and regulation of thrombosis, calcification and vascular function. Typical diabetic ROS and oxidative damage markers are shown in the red elliptic boxes. Green text boxes reflect the major therapeutic targets of current antidiabetic and cardiovascular therapies. NETs, neutrophil extracellular traps; NLRP3 inflammasome; HMGB1, high-mobility group box 1; VCAM-1, vascular cell adhesion molecule-1; IL, interleukin; TNF-*α*, tumor necrosis factor-*α*; CD68, cluster of differentiation 68 (macrosialin); 8-isoPG, 8-isoprostane; 8-oxoG, 8-oxoguanine; RAAS, renin-angiotensin-aldosterone system; SNS, sympathetic nervous system; ET-1, endothelin-1; mtROS, mitochondrial ROS; DAMPs, damage-associated molecular patterns; PGs, prostaglandins; ALDH-2, mitochondrial aldehyde dehydrogenase; 4-HNE, 4-hydroxynonenal; MDA, malondialdehyde; ACE, angiotensin-converting enzyme; AT_1_-receptor, angiotensin II type 1 receptor. Redrawn from Daiber et al^36^ and Steven et al^46^ under the terms and conditions of the Creative Commons Attribution license.

Major consequences linking suboptimally controlled DM to increased rates of CVD include glucotoxicity (advanced glycation end product [AGE]/RAGE signaling), inflammation, and oxidative stress, all of which contribute to micro- and macro-vascular damage, accelerated atherosclerosis, triggering thrombotic events, end-organ damage, and dysfunction.^31^, ^32^, ^33^ Importantly, these 3 pathophysiological pathways are linked (crosstalk concept) and can amplify each other.^34^, ^35^, ^36^, ^37^ As a functional readout of vascular damage, endothelial function represents an established marker, as it reflects the sum of endothelium-derived vasoactive signaling molecules that control vascular tone, blood flow, immune cell activity, and adhesion, thereby regulating blood pressure and perfusion. Endothelial dysfunction is an early diagnosable clinical marker of atherosclerosis^38^^,^^39^ and a predictor of CV events or mortality in patients,^40^^,^^41^ which can be determined in humans by 3 methods (flow-mediated dilation, plethysmography in peripheral vessels, or peripheral arterial tonometry).^33^ Diabetes mellitus is associated with endothelial dysfunction in humans.^42^ A population-based study (n = 631 participants) revealed that T2DM is associated with both endothelial dysfunction and low-grade inflammation, which explained, to a large extent, the higher CV mortality risk of diabetic subjects.^43^ Antioxidant interventions, such as supplementation with lipoic acid, vitamin C, or BH_4,_ improved endothelial dysfunction in diabetic subjects.^44^^,^^45^ Major components and concepts are summarized in Fig. 4.^36^^,^^46^

### B Glucotoxicity

Hyperglycemia leads to glucotoxicity via fueling of toxic metabolization pathways (eg, sorbitol formation)^47^^,^^48^ and the formation of AGE as well as the activation of their respective receptors (RAGE),^49^^,^^50^ all of which were prevented by the normalization of mitochondrial superoxide formation in hyperglycemic-cultured endothelial cells.^51^ Hyperglycemia leads to protein modifications, resulting in AGEs^52^ and contributing to endothelial dysfunction in preclinical studies.^53^ Signaling of AGEs via their receptors (RAGE) in diabetic rats triggers vascular complications via NOX-derived oxidative stress,^54^ mitochondrial reactive oxygen species (ROS) formation,^55^ and inflammation with atherosclerosis in mice.^56^ Macrophages from gp91phox null mice respond less efficiently to AGE stimulation.^54^ The diabetic phenotype, characterized by an increased expression of inflammatory cytokines and plasminogen activator inhibitor-1 (PAI-1), is likely influenced by enhanced protein kinase C activity, hexosamine metabolism, and sorbitol production through the polyol pathway.^57^ Blockade of AGE/RAGE signaling is also discussed as a novel therapeutic target in diabetic patients to prevent CV and renal complications.^58^^,^^59^ A meta-analysis found that higher soluble RAGE levels were associated with increased risk for CV events in T1DM patients.^60^

### C Oxidative stress, inflammation, and endothelial dysfunction

Oxidative stress is an accepted trigger of endothelial dysfunction in human subjects with prognostic implications^61^ and an initiator of atherosclerosis,^62^ although the oxidative loss of nitric oxide-mediated vasodilation can be also compensated by vasodilatory actions of hydrogen peroxide formation due to the presence of multiple CV comorbidities in a swine model.^63^ Large clinical trials support the role of oxidative stress in CV prognosis.^64^ There is also a well-documented role of oxidative stress in CV complications in diabetic individuals and animal models^65^ and T2DM patients.^66^^,^^67^ Oxidative stress in patients and animals with the comorbidities diabetes and hypertension also promotes systemic inflammation.^68^ Inflammation is an independent CV risk factor in CVD patients.^69^^,^^70^ Specific targeting of inflammatory pathways (eg, interleukin-1*β* blockade with canakinumab, and CANTOS trial) improved CV outcomes in patients with a history of MI.^71^ It is widely accepted that DM is associated with low-grade inflammation, facilitating endothelial (vascular) dysfunction and the development of atherosclerosis in human subjects.^72^ A meta-analysis of 23 trials comprising 1523 T2DM patients found decreased tumor necrosis factor-*α* and C-reactive protein (CRP) levels upon antioxidant therapy with omega-3 fatty acids.^73^ Mechanistically, the benefits of suppressing low-grade inflammation are probably due to improved macro- and micro-vascular function, as extensively reviewed elsewhere.^35^^,^^74^^,^^75^ Endothelial function is linked to the markers of oxidative stress, inflammation, obesity, and CV risk (eg, CRP, adiponectin, and brain natriuretic peptide).^33^ Endothelial dysfunction is widely found in diabetic subjects and can be normalized by antioxidant treatments.^44^^,^^45^ A population-based study found that T2DM is associated with both endothelial dysfunction and low-grade inflammation, explaining at least in part the approximately 43% of the increase in CV mortality risk in the setting of diabetes.^43^

### D Standard cardiovascular therapy against oxidative stress, inflammation, and endothelial dysfunction

Human evidence supports that CV drugs like angiotensin-converting enzyme inhibitors, type 1 angiotensin II receptor antagonists, and statins have indirect antioxidant properties, such as inhibiting NOX enzymes and preventing eNOS uncoupling.^76^^,^^77^ They increased the bioavailability of **^•^**NO by decreasing bradykinin breakdown and activating the B2 receptor, as shown in porcine arteries.^78^ They also prevent the activation of the phagocytic and vascular NOX2 enzyme, thus reducing cellular superoxide, hydrogen peroxide, and peroxynitrite levels in an experimental model of idiopathic-dilated cardiomyopathy^79^ by interfering with AT1-receptor-mediated diacylglycerol formation and protein kinase C activation.^80^ These drugs also have anti-inflammatory effects by interfering with monocyte adhesion and improving adjuvant arthritis severity in rats.^81^ Statins target the same pathophysiological parameters, reducing vascular inflammation and atherothrombosis in patients with CVD.^82^, ^83^, ^84^ As shown in preclinical models, they reduce the NADPH oxidase activity^85^^,^^86^ and improve the vascular bioavailability of ^•^NO.^87^ Moreover, statins activate the antioxidant and anti-inflammatory transcription factor NRF2 in animal models^88^^,^^89^ and mobilize endothelial progenitor cells in humans.^90^ Statins can also interfere with cardiotoxicity induced by other CV drugs.^91^

## III Targeting cardiovascular pathophysiology in type 2 diabetes mellitus

### A Targeting of cardiovascular pathophysiology by glucagon-like peptide 1 receptor agonists and dipeptidyl peptidase 4 inhibitors

#### 1 Dipeptidyl peptidase 4 inhibitors and glucagon-like peptide 1 receptor agonists in atherosclerosis

**Fig. 5:**
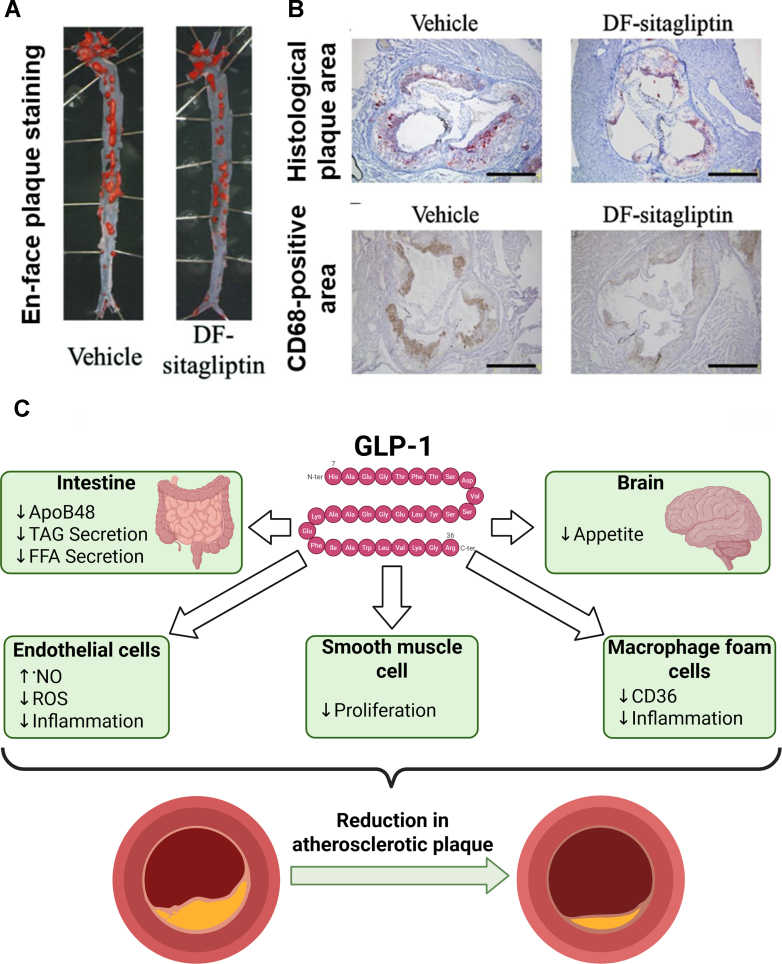
DPP-4 inhibition reduces atherosclerotic lesion formation and the number of macrophages within plaque in ApoE-deficient mice. (A) The relative surface area of the atherosclerotic lesion in vehicle-treated and sitaglitpin-treated ApoE-deficient mice. (B) Histological analyses of atherosclerotic lesion area (Oil Red O stain, upper panel) and the number of macrophages in plaque (anti-CD68 stain, lower panel) in aortic sinuses of vehicle-treated and des-fluoro-sitagliptin (DFS)–treated ApoE-deficient mice. Scale bars = 500 *μ*m. Reproduced from Matsubara et al^92^ with permission. (C) Proposed antiatherosclerotic properties of GLP-1 analogs. Modified from Ussher and Drucker^93^ with permission.

Matsubara et al studied the effects of the DPP-4i sitagliptin on atherosclerosis in mice,^92^ demonstrating the reduction of atherosclerotic lesions, improved endothelial function, and reduced infiltration of CD68^+^ cells into the vascular wall and expression levels of cytokines in ApoE^−/−^ mice (Fig. 5).^92^^,^^93^ Likewise, Shah et al found that alogliptin reduced chemotaxis and monocyte activation in LDLr^−/−^ and ApoE^−/−^ mice models of atherosclerosis.^94^ The administration of GLP-1R-activating GLP-1 peptide fragments for 12 weeks also showed antiatherosclerotic effects, reducing vascular inflammation and thus increasing plaque stability,^95^ and improving endothelial function in ApoE^−/−^ mice.^96^ In ApoE^−/−^ mice and LDLr^−/−^ mice, liraglutide and semaglutide treatment significantly attenuated plaque lesion development, in part independent of body weight and cholesterol-lowering.^97^ The beneficial effects of glucagon-like peptide 1-receptor agonists (GLP-1RA) in reducing oxidative stress may be based in part on inhibition of NOX2 in inflammatory monocytes of hypertensive mice, which trigger endothelial dysfunction.^98^ Recent studies show reduced oxidative stress in human monocytes after exendin-4 incubation, increased antioxidant capacity,^99^ and anti-inflammatory effects due to the modulation of NF-B-activity via PKA- and cAMP-dependent signaling pathways at the preclinical level.^100^ GLP-1RA supplementation and DPP-4 inhibition improve vascular function in animal models of atherosclerosis by reducing inflammation and oxidative stress. These effects are mediated by the GLP-1 receptor and DPP-4, respectively, expressed on endothelial cells. Preclinical studies support that GLP-1RA treatment (exendin-4 and active GLP-1 peptide fragments) improves endothelial ^•^NO synthase function, reduces cell activation via PI3 kinase/Akt-signaling pathway,^101^ and activates cAMP/PKA-signaling and cGMP signaling pathways,^102^^,^^103^ both in a GLP-1R-dependent and -independent manner. DPP-4 inhibitors like alogliptin and linagliptin also show potent vasodilatory effects involving Akt, eNOS, and ^•^NO formation in animal models.^104^^,^^105^ Additionally, GLP-1RA inhibit the expression of NOX subunits in human vascular endothelial cells.^106^ The potent atherosclerotic effects of GLP-1RA therapy were extensively reviewed previously^93^^,^^106^, ^107^, ^108^ and at the individual level of the endothelial, immune, and smooth muscle cells.^109^^,^^110^

#### 2 The antioxidant and anti-inflammatory profile of dipeptidyl peptidase 4 inhibitors and glucagon-like peptide 1 receptor agonists

Oeseburg et al found that DPP-4 inhibition protects against oxidative stress-induced DNA damage and cellular senescence in Zucker Diabetic Fatty rats, also by protein kinase A activation of antioxidant enzymes.^111^ They suggest that elevated GLP-1 is responsible, as the effects can be blocked by exendin fragment 9-39, a GLP-1 receptor antagonist. Other studies have also established reduced oxidative stress under DPP-4 inhibitor therapy in animal models of various diseases such as diabetes,^112^, ^113^, ^114^ cardiac I/R-injury,^115^ MI,^116^ abdominal aortic aneurysm,^117^ Parkinson disease,^118^ neurological disease,^119^ and sepsis.^105^^,^^120^ Sitagliptin prevented the diabetes-induced imbalance between ROS production and antioxidant defense, thereby ameliorating diabetic retinopathy in db/db mice.^121^ Human studies support the antioxidant effects of DPP-4 inhibition, for example, by reduced oxidative stress markers.^122^^,^^123^ Importantly, animal data provide evidence for glucose-independent reduction of oxidative stress by DPP-4 inhibition. DPP-4 binds to the adenosine deaminase enzyme, thereby regulating the enzyme’s activity and immunomodulatory functions in a GLP-1-independent manner, as shown in preclinical studies.^124^^,^^125^ DPP-4 targets other proteins involved in inflammation regulation such as caveolin-1, CXCR4, CXCL12 (SDF-1), and fibronectin,^126^ and immunomodulation is critical for the antioxidant properties of DPP-4i. According to a meta-analysis DPP-4 inhibition in humans increases the risk of infections like nasopharygitis and urinary tract infections, reflecting its immunomodulatory effects.^127^ In addition, recent evidence indicates that plasma levels of DPP-4 activity and soluble DPP-4 are dissociated from inflammation in mice and humans.^128^

Differentiating between DPP-4 inhibition and GLP-1-receptor-mediated effects is challenging due to the impact on various cell types and tissues. Most studies lack experiments with GLP-1 receptor inhibition, preventing differentiation between DPP-4 and GLP-1-dependent effects. Future research should focus on cell-specific GLP-1 receptor knockout animals. At least 1 study showed that liraglutide treatment of mice with arterial hypertension normalized blood pressure, cardiac hypertrophy, vascular fibrosis, endothelial dysfunction, oxidative stress, and vascular inflammation in a GLP-1R-dependent manner.^129^ Molecular proof was provided by using global GLP-1R knockout mice (*Glp1r*^*−/−*^), as well as endothelial cell-specific (Glp1r^flox/flox^xCdh5^cre^) and myeloid cell-specific knockout mice (Glp1r^flox/flox^xLysM^cre^), which blunted the protective effects of liraglutide, such as S-glutathionylation as a marker of eNOS uncoupling and increased NO bioavailability.

**Fig. 6:**
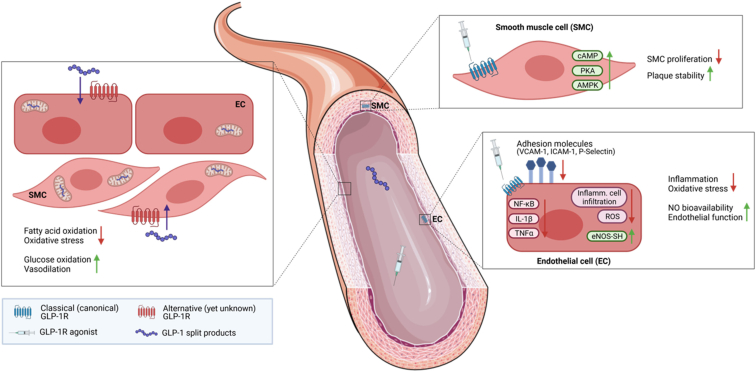
Proposed cellular targets and protective effects of GLP-1 receptor agonists (GLP-1RA) and GLP-1 split products in the vasculature. The benefits are likely mediated by a combination of GLP-1 receptor-dependent mechanisms on endothelial and smooth muscle cells (ECs, SMCs) and GLP-1 receptor-independent actions of GLP-1 metabolites. The existence of a second, yet not discovered, GLP-1 receptor has been proposed. The figure was created with BioRender (https://biorender.com) and by using elements from Servier Medical Art (https://smart.servier.com), which is licensed under a Creative Commons Attribution 3.0 Unported License (https://creativecommons.org/licenses/by/3.0/). Adopted from Helmstadter et al^134^ with permission.

In patients with T2D, reduced GLP-1 levels were associated with enhanced oxidative stress markers and a higher risk for CV events,^130^ and GLP-1RA therapy ameliorated arterial stiffness, left ventricular myocardial deformation, and oxidative stress.^131^ AMPK regulates oxidative stress in endothelial cells,^132^ and activation via GLP-1 receptor signaling reduces NOX-dependent oxidative stress in cardiomyocytes by AMPK activation.^133^ A summary of the beneficial actions of GLP-1RA to prevent oxidative stress and inflammation in smooth muscle and endothelial cells and improve vascular function in various inflammatory diseases is shown in (Fig. 6).^134^ There, GLP-1R-mediated effects protect against smooth muscle cell proliferation, increase plaque stability, suppress inflammation and oxidative stress, and improve nitric oxide bioavailability and endothelial function.^134^ Liraglutide reduced the markers of inflammation, leukocyte rolling on the endothelium, and infiltrating proinflammatory myeloid Ly6G^−^Ly6C^+^ and Ly6G^+^Ly6C^+^ cells into the vascular wall of hypertensive mice.^129^

Also antifibrotic effects of GLP-1RA may play a role, as shown in patients with metabolic dysfunction-associated steatohepatitis (MASH, previously termed “nonalcoholic” steatohepatitis) where liraglutide decreased the markers of fibrosis.^135^ In a mouse model, GLP-1 prevented aortic banding-dependent myocardial fibrosis by the inhibition of mTOR pathway and improved autophagy.^136^ Suppression of paracrine communication between infiltrated macrophages and fibroblasts by exendin-4, preventing cardiac fibroblast differentiation in diabetic mice, represents another protective mechanism.^137^

#### 3 Gliptins and glucagon-like peptide 1 receptor agonists antithrombotic effects

**Fig. 7:**
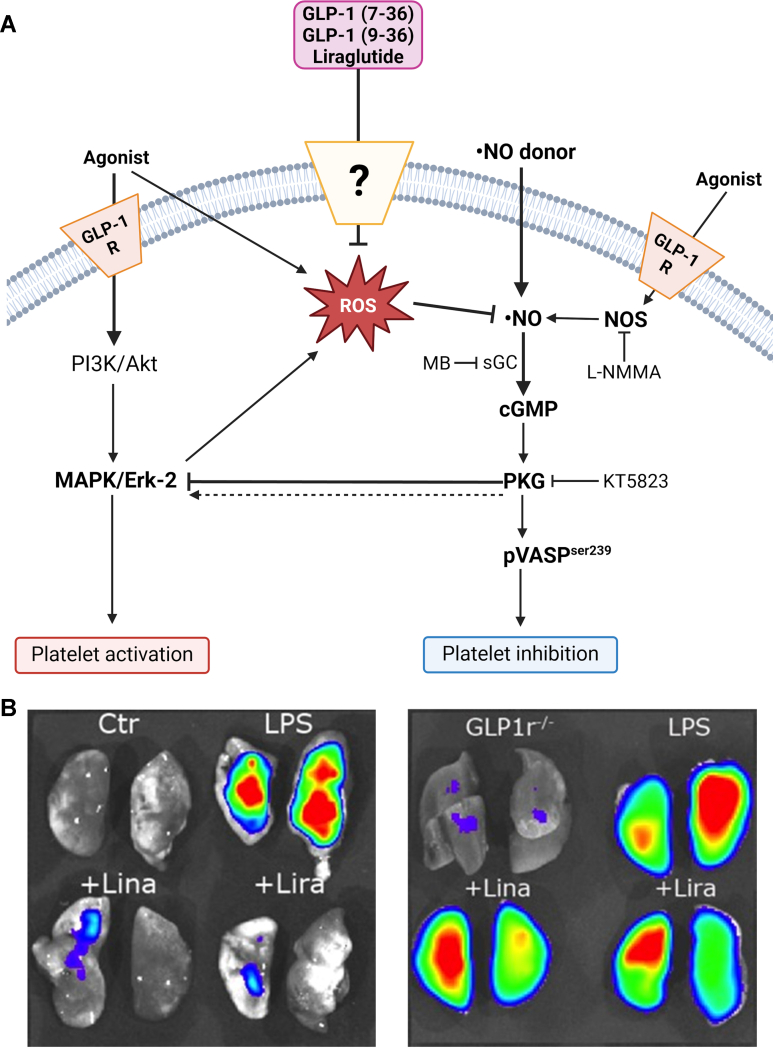
GLP-1 reduces platelet aggregation and thrombus formation. (A) The scheme shows the actions of GLP-1RA on platelet function by improving the NO inhibitory effects and attenuating, via a PKG-me-diated mechanism, the activation of PI3-K and MAPK signaling stimulated by agonists. In this scenario, GLP-1 may interfere with the stimulant-induced ROS production, and all these effects are independent of the known GLP-1 receptor (GLP-1R), which is expressed on the platelet surface. Adopted from Barale et al^138^ with permission. (B) Microvascular thrombosis was detected by fluorescence imaging using fluorescent microbeads in endotoxemic wild-type mice with linagliptin and liraglutide treatment and *GLP1r*^*−*/*−*^ mice. Representative images of the lungs are shown. Endotoxemia by LPS was associated with microvascular thrombosis as reflected by increased fluorescence signal in the lungs, which was prevented by linagliptin and liraglutide treatment. The protective effects were absent in *GLP1r*^*−*/*−*^ mice. Adopted from Steven et al^141^ with permission.

GLP 1R stimulation has been linked to an inhibitory effect on platelets, with preclinical studies showing antiaggregation effects on murine and human platelets^138^^,^^139^ and reduced clot formation in rodents.^140^^,^^141^ However, the expression of GLP-1R on platelets is debated, with some studies confirming it on the mRNA level,^140^ whereas others do not.^139^ Positive GLP-1R protein expression was observed in both species,^138^^,^^141^ but the specificity of various GLP-1R antibodies remains uncertain. The antithrombotic and antiaggregatory effect of native GLP-1 and exenatide was accompanied by cAMP generation, which was absent in *eNOS*^*−*/−^ mice^140^ and PKA-dependent phosphorylation of the vasodilator-stimulated phosphoprotein at serine 157 in murine and human platelets, which was abolished in global *Glp1r*^*−*/*−*^ mice.^141^ It was proposed that GLP-1R activation on platelets may activate platelet eNOS (via cAMP/PKA/phospho-eNOS at Ser1177), and platelet-derived NO, conveying the antithrombotic effects (mechanisms summarized in Fig. 7A).^138^^,^^141^^,^^142^ However, another ex vivo study using isolated murine cells reported that native GLP-1 prevents thrombus formation under physiological flow conditions independent of a platelet GLP-1R,^139^ possibly invoking the contribution of GLP-1R on other cells in vivo, for example, the endothelium. We found that the aggregatory potential of platelets (endogenous thrombin potential) was diminished in *Dpp4*^*−*/*−*^ mice and exacerbated in *Glp1r*^*−*/*−*^ mice.^141^ In the same study, a time-dependent decrease of platelet count in the whole blood of endotoxemic mice correlated with increased microthrombus formation in the lungs (and other organs), and this disseminated intravascular coagulation, a significant complication in septic shock, was prevented by linagliptin and liraglutide treatment (Fig. 7B). Still, protection was abolished in *Glp1r*^*−*/*−*^ mice. Importantly, suppression of platelet aggregation, microthrombi formation, and markers of oxidative stress and inflammation by the drugs and DPP-4 knockout also improved the mortality of septic animals. The preclinical data are supported by small cohort human studies reporting diminished platelet aggregability in T2DM patients treated with liraglutide,^143^ exenatide,^144^ and GLP-1RA in general.^145^ Similarly, acute liraglutide treatment attenuated thromboxane-induced aggregation of human platelets ex vivo, obtained from subjects with aspirin exacerbated respiratory disease.^146^

#### 4 Glucagon-like peptide 1 receptor agonists: a therapeutic approach for mitigating myocardial ischemia/reperfusion injury

**Fig. 8:**
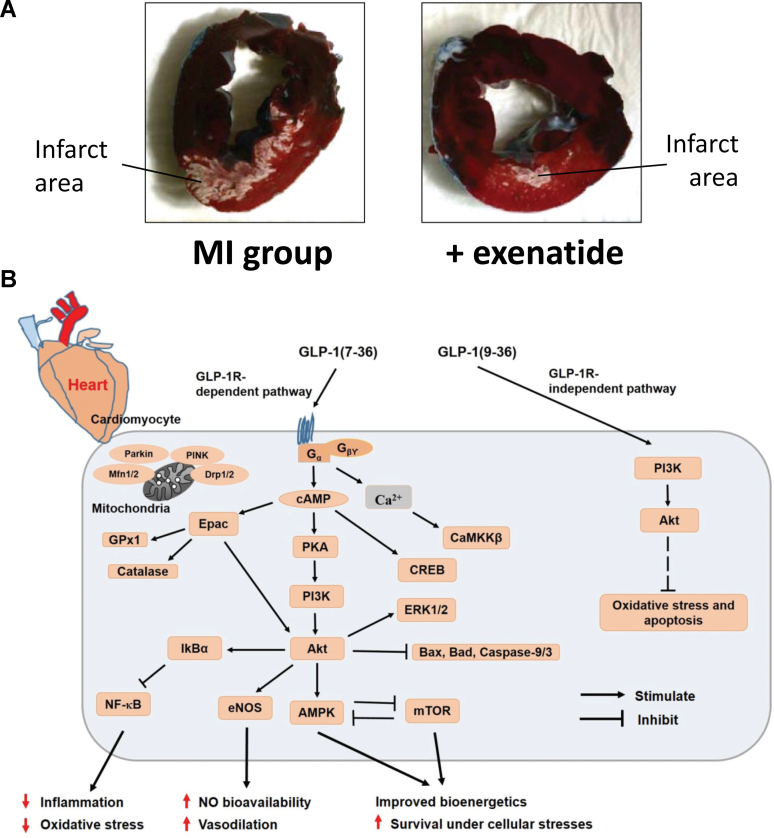
GPL-1R agonist therapy reduces myocardial infarction damage in a porcine model. (A) Representative pictures after Evans Blue and triphenyltetrazolium chloride staining reflecting myocardial infarct size are shown. **Blue** indicates non-threatened myocardium, **red** indicates the noninfarcted area within the area at risk, and **white** indicates myocardial infarction area with tissue damage. Reproduced from Timmers et al^151^ with permission. (B) Schematic diagram representing cardioprotective molecular mechanisms of GLP-1 analogs in cardiomyocytes. The GLP-1R-dependent and -independent signaling pathways have been described as contributing to healthy myocardium and heart functions under pathophysiological conditions. The binding of GLP-1 to its receptor activates adenylate cyclase, and cAMP levels are elevated, which is followed by the activation of downstream cAMP sensitive molecular effectors in a GLP-1R-dependent way. Although GLP-1R-independent GLP-1(9-36) activates PI3K, Akt, and the downstream signaling pathway, the full mechanisms of this process are not yet known. Akt, protein kinase B; AMPK, AMP-activated protein kinase; CaMKK*β*, calcium ions/calmodulins dependent protein kinase kinases *β*; cAMP, cyclic adenosine monophosphate; CREB, cAMP-response element binding protein; Drp, dynamin-related protein; eNOS, endothelial nitric oxide synthase; Epac, exchange protein activated by cAMP; ERK, extracellular-signal-regulated kinase; GLP-1, glucagon-like peptide-1; Mfn, Mitofusin; mTOR, mammalian target of rapamycin; NF-κB, nuclear factor-κB; PI3K, phosphatidylinositide 3-kinase; PINK1, PTEN-induced kinase 1. Reproduced from Pandey et al^153^ with permission.

Acute myocardial infarction (AMI) is a leading cause of global morbidity and mortality, with 8.9 million deaths reported in 2020.^147^ Although advancements in reperfusion strategies, such as percutaneous coronary intervention, have improved survival rates, MI/RI remains a significant complication, contributing to cardiomyocyte death and adverse outcomes.^148^ Beyond reperfusion, pre/post/remote-conditioning and pharmacological activation of autophagy and prevention of different forms of cell death may represent alternative therapeutic options.^149^^,^^150^ MI/RI involves a complex interplay of oxidative stress, inflammation, and calcium overload, which are exacerbated in diabetic patients, who show worse short- and long-term prognoses following AMI. Key findings suggest lowering infarct size by exenatide in a porcine model of I/R (Fig. 8A).^151^ Emerging research highlights GLP-1RA as promising agents to mitigate MI/RI.^152^

During MI, the oxidative capacity of mitochondria is impaired, leading to inadequate ATP production and a shift toward inefficient glycolysis.^154^ GLP-1RA enhance glucose oxidation and suppress fatty acid oxidation, optimizing energy utilization in ischemic cardiomyocytes. For instance, GLP-1 facilitates a metabolic switch that reduces oxidative stress while maintaining myocardial energy levels.^155^ In animal models, GLP-1 improved anaerobic glycolysis and decreased myocardial hypoxia, effectively reducing infarct size and enhancing recovery.^156^

A key cardioprotective mechanism of GLP-1RA is activating the reperfusion injury salvage kinase (RISK) pathway during myocardial reperfusion. The RISK pathway involves kinases such as PI3K/Akt, ERK1/2, and MAPK, which prevent the opening of mitochondrial permeability transition pores, thereby inhibiting cell apoptosis and necrosis.^157^ Additionally, GLP-1 reduces ROS generation through the cAMP/Epac and PI3K/Akt pathways, improving mitochondrial function and reducing myocardial injury as shown for cardiomyocytes and cardiomyoblasts.^158^^,^^159^

Inflammation plays a critical role in exacerbating MI/RI. GLP-1RA inhibit the infiltration of inflammatory cells and the production of proinflammatory factors. Liraglutide, for example, activates AMPK and eNOS, promoting nitric oxide synthesis and reducing leukocyte adhesion. Furthermore, GLP-1RA suppress inflammasome activation, such as NLRP3 and caspase-1, thereby reducing pyroptosis and myocardial damage. By mitigating inflammation, GLP-1RA helps preserve myocardial function and prevents adverse cardiac remodeling.^158^^,^^159^

Calcium overload significantly contributes to MI/RI, leading to ROS generation and cardiomyocyte death. GLP-1RA regulate intracellular calcium levels by stabilizing sarcoplasmic reticulum calcium stores and maintaining ATP production. Liraglutide prevents Ca^2+^-mediated oxidative stress and reduces arrhythmogenic calcium leakage in rat hearts, improving cardiac outcomes during reperfusion.^160^ Clinical trials have validated the cardioprotective effects of GLP-1RA. For instance, liraglutide has been shown to reduce infarct size and improve systolic function in ischemic models.^161^ Overall, GLP-1RA normalize kinase signaling in cardiomyocytes during MI/RI conferring improved survival by suppression of inflammation, oxidative stress, and apoptosis and normalization of nitric oxide bioavailability and bioenergetics (Fig. 8B).^153^^,^^162^ Modulation of cardiac ventricular excitability by GLP-1R-mediated stimulation of cardiac parasympathetic (vagal) neurons may also add to the overall cardioprotective effects of GLP-1RA.^163^

Similarly, semaglutide reduced myocardial hypoxia and oxidative stress, offering robust protection against MI/RI in a rat model.^164^ These findings are particularly significant in diabetic patients, who are more vulnerable to MI/RI due to heightened oxidative stress and metabolic dysfunction. GLP-1RA represent a novel therapeutic avenue for addressing MI/RI in both diabetic and nondiabetic populations. By targeting multiple pathological pathways, these agents offer a comprehensive approach to reducing myocardial injury and improving post-AMI outcomes. Further research is needed to optimize dosing strategies and investigate their integration into standard MI/RI management protocols.

### B Targeting of cardiovascular mechanisms by sodium-glucose cotransporter 2 inhibitors

#### 1 Antioxidant, anti-inflammatory, and vasculoprotective effects of sodium-glucose cotransporter 2 inhibitors in type 1 diabetes

**Fig. 9:**
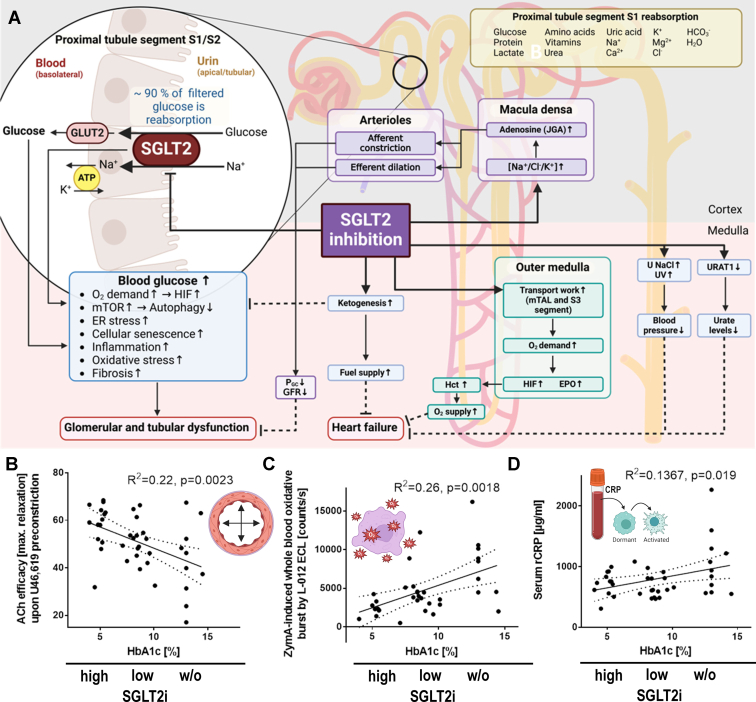
Proposed mechanisms of SGLT2 inhibitor-conferred protection in T2DM. (A) Left: detailed mode of action of SGLT2i. Summarized and redrawn from previous work.^170^^,^^171^ Right: **(**1) the normalization of the glycemic condition and prevention of glucotoxicity (AGE/RAGE signaling), (2) reduction of oxidative stress and inflammation and (3) improvement of endothelial function. Besides the direct protective effects of empagliflozin by glucose-lowering, indirect epigenetic/pleiotropic effects may be active leading to reduced cardiovascular risk as shown by the EMPA-REG trial. Linear regression analysis for correlations between HbA1c and endothelial function (ACh efficacy, B), zymosan A-induced whole blood oxidative burst (C) and serum CRP levels (D) using a total of 35-41 rats. The 3 groups of data points indicate the untreated and low and high-dose empagliflozin groups. B-D were adopted from Steven et al^46^ with permission.

Current antidiabetic treatments lose efficacy over time, leading to disease progression.^165^ This is due to the diminished insulin-secreting capacity of *ß*-cells and the desensitization of insulin receptor-dependent pathways. SGLT2 inhibitors, which do not depend on insulin, have shown beneficial effects in DM type II and type 1 diabetic patients.^166^^,^^167^ Empagliflozin treatment in rats with streptozotocin-induced type 1 diabetes dose-dependently reduced blood glucose levels and oxidative stress in aortic vessels and normalized endothelial function by improved ^•^NO/cGMP signaling.^168^ The study also found that SGLT2i therapy reduced oxidative burst in whole blood and reversed proinflammatory phenotype and glucotoxicity via AGE/RAGE signaling in diabetic animals. These findings follow a previous study on preventing low-grade inflammation in STZ rats by antioxidant therapy.^169^ However, the study’s limitation is that STZ treatment kills only some part of islet cells directly, and empagliflozin therapy preserves and improves insulin secretion. This differs from human T1DM, where all islet cells are lost due to autoimmune-driven killing. The mode of action and the summary of the beneficial actions of SGLT2i in T1DM and T2DM models are shown in Fig. 9A.

#### 2 Antioxidant, anti-inflammatory, and vasculoprotective effects of sodium-glucose cotransporter 2 inhibitors in type 2 diabetes mellitus

A study using Zucker Diabetic Fatty (ZDF) rats and lean controls as models for T2DM found that empagliflozin treatment restored glycemic control by preserving *ß*-cell integrity and function, as well as safeguarding *α*-cells within pancreatic islets and their glycogen content.^37^ Empagliflozin also effectively prevented glucotoxicity, precisely methylglyoxal accumulation, AGE formation, and RAGE-dependent signaling. Methylglyoxal is toxic to tissue and plays a role in diabetic neuropathy development and progression.^172^ In diabetic conditions, increased AGE/RAGE signaling contributes to oxidative stress and vascular complications via the activation of NADPH oxidases^173^ and impairment of ^•^NO/cGMP signaling at the preclinical level.^54^ Amelioration of oxidative stress parameters in T2DM patients by SGLT2i therapy was supported by a study demonstrating decreased DNA oxidation (8-oxodG) after 12 weeks of dapagliflozin treatment.^174^ This is in line with key findings of the EmDia trial that empagliflozin improves diastolic function in patients with T2DM and elevated end-diastolic pressure^175^ and ameliorates DNA oxidation (unpublished EmDiaOx trial, Daiber et al).

Empagliflozin treatment also improved endothelial function by improving ^•^NO/cGMP signaling, reducing oxidative stress, and preventing inflammation in diabetic ZDF rats via suppression of AGE/RAGE signaling.^46^ This is due to the prevention of adverse phosphorylation of eNOS at Thr495 by protein kinase C and of oxidative inactivation of the soluble guanylyl cyclase and oxidative depletion of ^•^NO and the eNOS cofactor BH_4_ (reviewed in Daiber et al^33^). ChIP analysis also revealed epigenetically prevented inflammation and AGE/RAGE signaling.^46^ In addition, a significant inverse correlation between endothelial function and HbA1c was identified, which was positively correlated with leukocyte-dependent oxidative burst and CRP levels (Fig. 9B-D),^46^^,^^170^^,^^171^ further supporting that AGE/RAGE signaling triggers low-grade inflammation.^56^ Empagliflozin’s anti-inflammatory properties may be due to the efficient suppression of AGE/RAGE signaling and inhibition of inflammatory pathways. However, decreased histone3 lysine4 dimethylation in kidney promoter regions and subsequent downregulation of *iNOS*, *IFNγ*, and *RAGE*^46^ may also impact the effects. Thus, the study cannot differentiate whether these effects are due to improved glycemic control or a specific property of empagliflozin. Since SGLT2i failed to improve hyperlipidemia and hyperinsulinemia in this study, a combination therapy with SGLT2i and lipid-lowering drugs may be promising in T2DM and metabolic syndrome. Antidiabetic SGLT2i have been investigated for treating MAFLD/MASH.^176^ Empagliflozin has improved liver fibrosis and steatosis markers in MAFLD patients^177^^,^^178^ and ameliorated MASH phenotype in mice.^179^ The cardio-metabolic-renal benefits of SGLT2i have been thoroughly reviewed.^180^

#### 3 Pleiotropic cardiovascular effects

**Fig. 10:**
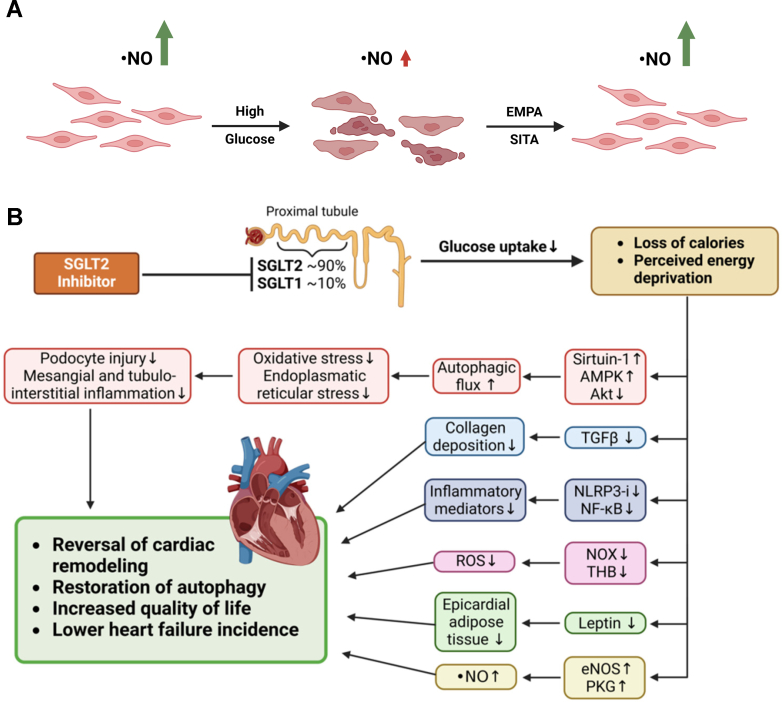
Pleiotropic beneficial effects by SGLT2i in cultured endothelial cells and hypothetical pharmacological mechanisms. (A) Empagliflozin (EMPA) and the dipeptidyl peptidase-4 (DPP-4) inhibitor sitagliptin (SITA) prevented cell death in cultured human endothelial cells (HUVECs) under hyperglycemic conditions. In addition, both drugs protected eNOS function and nitric oxide formation in the hyperglycemic cells.Summarized from previous work.^46^ (B) Proposed mechanisms of SGLT2i-conferred pleiotropic effects. Summarized and redrawn from previous work.^190^^,^^191^

General support for the pleiotropic effects of SGLT2i is based on studies demonstrating cardioprotective effects of SGLT2i in nondiabetic mouse models of ischemia/reperfusion.^181^, ^182^, ^183^ It was also reported that SGLT2i confers cardioprotection and reduces myocardial infarct size by increasing parasympathetic activity in animal tissues.^184^ In vitro studies showed pleiotropic improvements in viability and eNOS function in hyperglycemic human umbilical vein endothelial cells (HUVECs) following SGLT2i treatment—independent of glycemic control (Fig. 10A).^46^ However, the experiments were limited by the suprapharmacological concentrations used and the lack of knowledge about SGLT2's presence and function in endothelial cells. The effects of empagliflozin are considered “pleiotropic” until further investigation is conducted. The RAGE inhibitor FPS-ZM1 demonstrated a protective effect against hyperglycemia-induced complications, but further investigation is needed to determine its mechanism. Strong evidence for these off-target effects of SGLT2i comes from a report on reduced infarct size independent of the cotransporter by a study using *Sglt2* knockout mice.^185^ Besides the widely accepted pleiotropic mechanisms of SGLT2i, for example, improved eNOS activity, nitric oxide bioavailability, diminished ROS formation, and decreased inflammation, STAT3 upregulation also seems to play a role in endothelial cell protection.^186^ STAT3 activation by SGLT2i was shown to improve microvascular endothelial cells and reduce oxidative stress, decreasing myocardial infarct size in nondiabetic mice.^182^ Others have extensively reviewed the pleiotropic effects (summarized in Fig. 10B), especially the off-target CV benefits.^187^, ^188^, ^189^, ^190^, ^191^

Pleiotropic effects of SGLT2i were also observed in cell types other than endothelial cells. Dapagliflozin increases the activity of neurons in the central nervous system with beneficial effects on CV activity.^192^ Improved autonomic control is mediated by the inhibition of central or peripheral SGLT2, thereby suppressing sympathetic activity and conferring direct blood pressure-lowering effects. In line with this, a study in rats found that SGLT2i-mediated arterial relaxation depends on calcitonin gene-related peptide increases upon the stimulation of neurons in sensory nerves by SGLT2i, independent of glucose transport.^193^ Also, direct effects of SGLT2i on smooth muscle cells and cardiomyocytes became evident since higher SGLT2 expression in human microvessels and hearts is associated with low-grade inflammation, stimulating the AT_1_-receptor/NADPH oxidase axis with impaired eNOS function and nitric oxide bioavailability.^194^ Suppression of smooth muscle cell proliferation by SGLT2i in different models in vivo^195^ and ex vivo^196^^,^^197^ further supports the direct actions of the drugs. Delayed ischemic damage by empagliflozin was associated with inhibition of sodium-hydrogen exchanger activity, which was also observed in isolated cardiomyocytes.^198^ Empagliflozin improved diastolic function in pigs with heart failure via decreased myocardial fibrosis and higher nitric oxide and cGMP levels, also reflected by better relaxation of isolated cardiomyocytes from empagliflozin-treated animals.^199^ In cultured rat cardiomyocytes stimulated with high glucose and palmitic acid, SGLT1 inhibition by the least selective SGLT inhibitor canagliflozin prevented fibrosis and apoptosis and impaired mitochondrial function.^200^ The antifibrotic effects of dapagliflozin were independent of SGLT1/2 inhibition and glucose-lowering effects, as found by the studies of isolated cardiofibroblasts and diminished collagen content^201^ and suppression of angiotensin-II-dependent cardiac fibrotic remodeling by suppressing the TGF-*β*1/Smad pathway in rats.^202^

As mentioned above, empagliflozin changes the epigenetic landscape in diabetic rats, as shown by ChIP analysis supporting inhibitory regulation of inflammation and AGE/RAGE signaling^46^ as observed by others.^203^ Dapagliflozin was reported to modulate DNA methylation and gene expression in islet cells^204^ and suppress DNA damage and hypermethylation caused by hyperglycemia in murine cells.^205^ More recent animal studies also revealed that empagliflozin, sotagliflozin, and dapagliflozin improve confer cardio- and neuro-protective actions by positive actions on the gut-heart-brain axis and beneficial modulation of the microbiome.^206^, ^207^, ^208^ It was speculated that altered lipid profiles by SGLT2i affect the microbiota, which in turn causes changes in circulating microbial metabolites toward protective effects at the preclinical level.^209^^,^^210^ Suppression of thrombus formation by diminishing NOX-2-derived oxidative stress and markers of thromboinflammation was also reported for SGLT2i therapy in T2DM patients.^145^

##### a Critical remark

Importantly, CV outcome benefits of SGLT2i are not only reported in diabetic patients but also in nondiabetic patients with CVD. The benefits to patients with AMI are not clear or consistent so far.^211^ Although the EMMY study reported these benefits, the DAPA-MI study observed no benefit on mortality or heart failure hospitalization. The EMPACT-MI study described a mixed picture. In preclinical studies, the infarct site was reduced in mice, rats, and pigs without and with diabetes by different SGLT2i drugs. Surprisingly, the infarct size reduction rather required chronic oral pretreatment with SGLT2i inhibition and was observed in transgenic mice lacking the SGLT2 transporter.^185^ The mechanisms underlying these pleiotropic effects of SGLT2i are still enigmatic but may comprise sodium-proton exchange inhibition, antioxidant properties, protective ketone signaling, improved mitochondrial respiration, activation of the signal transducer and activator of transcription 3 (STAT3) activation, and inhibition of the inflammasome.^211^ A study by Nikolaou et al reported improved flow-mediated dilation in diabetic patients with STEMI by empagliflozin, suggesting improved coronary vascular function, which was also supported by the animal part of the study, where endothelial transcriptome dysregulation and impaired microvascular function after AMI were improved by SGLT2i, also correlating with reduced infarct size.^212^ Impaired coronary blood flow contributes to the progression of heart failure and therefore represents an attractive target for established and novel therapeutic regimens.^213^

## IV Modern therapy for type 2 diabetes mellitus

Patients with established CVD experience benefits beyond glucose control from new drugs like GLP-1RA or SGLT2i.^214^^,^^215^ The GLP-1RA liraglutide and semaglutide reduced death from CV causes, nonfatal MI, or nonfatal stroke compared with a therapy consisting of metformin and insulin (standard care).^216^^,^^217^ Similarly, the SGLT2i empagliflozin and canagliflozin reduced the rates of CVD outcomes, and empagliflozin, canagliflozin, and dapagliflozin reduced the rates of hospitalization for heart failure.^218^^,^^219^ Recommendations and guidelines already reflect evidence supporting the use of these new drugs, and diabetic patients are treated more individually according to their comorbidities.^215^ A comparison of the efficacy of different antidiabetic monotherapies and combination therapies is presented in Fig. 3. This review will focus on the 3 most recently approved classes of glucose-lowering medicines, DPP-4i, GLP-1RA, and SGLT2i therapy, describing the clinical properties and mechanisms of action.

Dipeptidyl peptidase-4 (DPP-4) is an exopeptidase, CD26, that cleaves N-terminal dipeptides from alanine and proline-rich proteins.^220^ It belongs to the DPP family, which includes DPP-1, DPP-6, DPP-9, quiescent cell proline dipeptidase (QPP), and fibroblast activation protein.^126^^,^^220^ DPP-4 is not only known for the degradation of incretins but also cleaves dozens of nonincretin peptides and acts as a chaperone with membrane-bound proteins.^126^ It is expressed on the surface of endothelial, epithelial, and immune cells in various tissues.^221^, ^222^, ^223^, ^224^ GLP-1 is an incretin hormone released from L-cells in the intestine, involved in glycemic control, and is an attractive target for diabetes treatment.^225^^,^^226^ It binds to its receptor, expressed on pancreatic *ß*-cells, cardiomyocytes, endothelial cells, and immune cells. GLP-1 increases cAMP levels and stimulates insulin release, whereas it reduces glucagon release through direct and indirect actions on pancreatic *α*-cells.^227^, ^228^, ^229^ GLP-1 is derived from the proglucagon gene and has a half-life of less than 2 minutes due to rapid degradation by DPP-4 and renal clearance.^230^^,^^231^ Two pharmacological strategies for enhancing GLP-1 action on glucose metabolism are inhibiting DPP-4 enzymatic activity by gliptins and the development of degradation-resistant GLP-1RA.

Several DPP-4 inhibitors (DPP-4i, for example, vildagliptin, alogliptin, sitagliptin, linagliptin, and saxagliptin) and GLP-1RA (eg, liraglutide, exenatide, dulaglutide, and semaglutide) have been approved by the European Medicines Agency for the treatment of T2DM. DPP-4i have beneficial effects on CVD in mouse models,^92^^,^^94^ hepatic steatosis in mice,^232^ and stroke in mice,^233^ although confirmation by large human cohort studies is yet missing. A human case report provided evidence for the beneficial effects of DPP-4i on the CV complications of psoriasis.^234^ These diseases are also highly related to oxidative stress and inflammation, suggesting antioxidant and anti-inflammatory effects of DPP-4i. DPP-4 inhibitors also improve the number and “homing” of endothelial progenitor cells in animal^235^^,^^236^ and human studies.^237^ However, validation of these concepts in human outcome studies is limited,^238^ and DPP-4i may not reduce inflammation in humans with CVD and T2DM to the same extent observed in preclinical studies.^128^

**Fig. 11:**
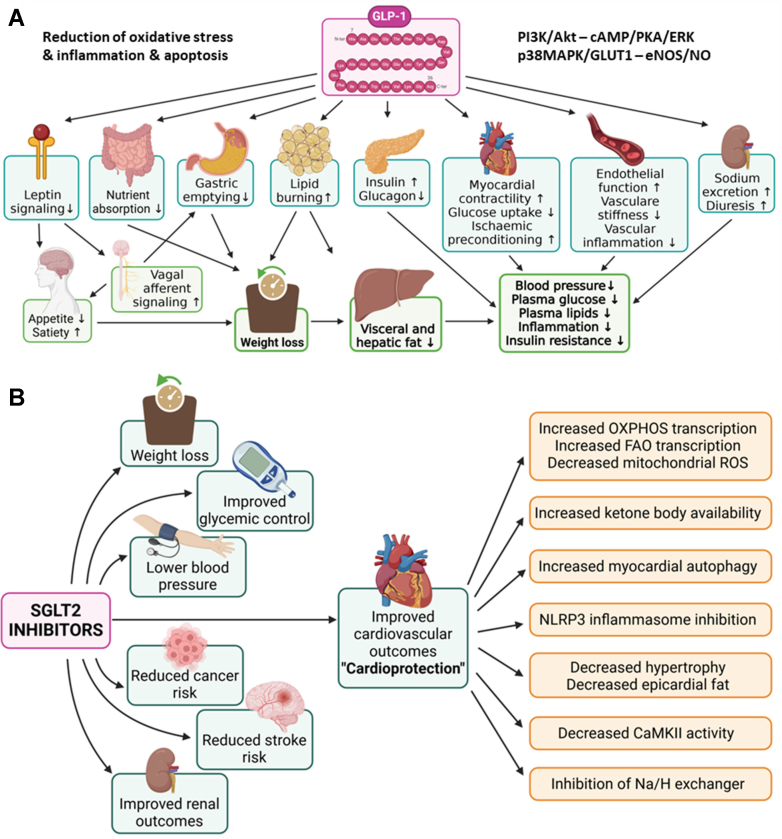
Major health effects and modes of action of (A) GLP-1 receptor agonists (partially shared by DPP-4 inhibitors) and (B) SGLT2 inhibitors. Summarized and redrawn from data in Ussher and Drucker,^93^ Boyle et al,^247^ and Yaribeygi et al^248^ (A) and Chen et al,^185^ Perry and Shulman,^249^ and Sawicki et al^250^ (B).

GLP-1RA may also modulate the activity of endothelial progenitor cells.^239^ The major health effects of DPP-4i and GLP-1RA are summarized in Fig. 11A. Beyond their actions on glucose control, some GLP-1RA are also approved for weight loss in people with overweight and comorbidities or obesity^240^ and CV risk reduction in people with T2DM and/or obesity,^108^ and approval for indications such as heart failure, metabolic liver disease, and osteoarthritis is pending.^241^ An overview on the drug development and clinical use of these agents is provided in.^242^^,^^243^ Mechanistically, the beneficial pleiotropic effects of GLP-1RA are based, to some extent, on central mechanisms that may be related to alteration of leptin signaling and loss of appetite as shown in animals.^244^ Central effects of GLP-1 are also mediated by the effects on vagal circuits impacting neurons of the dorsal motor nucleus of the vagus, as shown in rats.^245^ However, the central effects cannot explain all pleiotropic systemic effects of GLP-1RA, for example, specifically not those obtained ex vivo in isolated non-neuronal cells. Regarding GLP-1RA, there is still ongoing discussion on the use of short- versus long-acting drugs.^246^ Short-acting drugs are characterized by a shorter peak plasma concentration period, followed by intervals where plasma concentration falls below therapeutic levels, and long-acting drugs maintain a steadier state plasma concentration, with only minor fluctuations between doses. Due to these differences in pharmacokinetics, long-acting GLP-1RA are more efficient in terms of glucose-lowering (HbA1C reduction) and weight loss.

Gliflozins are inhibitors of sodium-glucose cotransporter 2 (SGLT2), which is responsible for reabsorption of >90% of glucose from primary urine, as shown in mice,^251^ and are widely approved for treating T2DM patients.^214^ SGLT2 inhibitors block renal glucose reabsorption, leading to increased urinary excretion of glucose. This prevents hyperglycemic episodes and glucotoxicity, preventing hyperglycemia-induced damage through AGE and receptor activation (RAGE) in preclinical studies.^168^^,^^252^ SGLT2i are insulin-independent, preventing deteriorating *β*-cell function and desensitization to insulin signaling,^165^ and reducing oxidative stress and inflammation by AGE/RAGE signaling as shown by preclinical research.^36^^,^^51^^,^^55^^,^^56^ Large-scale clinical trials have demonstrated that empagliflozin reduces CV and overall mortality in T2DM patients at high CV risk,^219^^,^^253^ actions shared by dapagliflozin^254^^,^^255^ and other SGLT2i.^214^ According to meta-analysis data, SGLT2i therapy also results in significant weight loss (an average difference of 2.3 kg at the highest dose), representing an additional protective effect,^256^^,^^257^ especially in obese patients with CVDs.^258^ In contrast, improvement in CV outcomes has not been demonstrated with DPP-4i. The significant health effects of SGLT2i are summarized in Fig. 11B.^247^, ^248^, ^249^, ^250^

### A Cardiovascular outcomes with glucagon-like peptide 1 receptor agonists: evidence from major trials

Like SGLT2i, GLP-1RA have been studied in 9 clinical trials investigating CV safety in people with T2DM and 1 in people with obesity. Data from cardiovascular outcome trials (CVOTs), including ELIXA, SUSTAIN-6, LEADER, HARMONY, REWIND, and AMPLITUDE-O, highlight the safety and CV benefits of GLP-1RA in patients with T2DM. Although ELIXA (lixisenatide) demonstrated CV safety without superiority in reducing major adverse cardiovascular events (MACE) in people with a history of an acute coronary event,^259^ trials such as LEADER (liraglutide) and SUSTAIN-6 (semaglutide) showed significant MACE reductions in people with established CVD.^216^^,^^217^^,^^260^ For example, LEADER reported a 22% reduction in CV mortality compared with placebo, underscoring the protective potential of GLP-1RA.^217^

REWIND, the largest GLP-1RA trial in T2DM to date, studied dulaglutide and its CV benefits.^261^ Over a median 5.4-year follow-up, dulaglutide reduced the composite outcome of nonfatal MI, nonfatal stroke, and CV death by 12%. These findings extended to patients with lower baseline HbA1c levels, suggesting that the benefits are independent of glycemic control. Similarly, AMPLITUDE-O revealed a 27% reduction in MACE with efpeglenatide, further supporting GLP-1RA efficacy across diverse patient populations.^262^ Structural and pharmacokinetic differences and/or trial design among GLP-1RA appear to influence outcomes. Additionally, the shorter follow-up duration in ELIXA (2.1 years) compared with trials like REWIND (5.4 years) may have contributed to the observed variability.^261^

Beyond MACE reduction, GLP-1RA impact CV biomarkers and secondary risk factors. Studies indicate improvements in blood pressure, lipid profiles, and inflammation in T2DM patients.^263^ Mechanistically, GLP-1RA may induce vasodilation and enhance sodium excretion, potentially reducing atherosclerotic burden. Preclinical data from ApoE-deficient mouse models demonstrated that liraglutide improved plaque stability, reduced endothelial dysfunction, and attenuated weight gain in advanced atherosclerosis.^264^ GLP-1RA also confer antihypertensive effects,^265^^,^^266^ potent renoprotection in T2DM patients with chronic kidney disease,^267^ and neuroprotection against ischemic stroke.^268^^,^^269^ The beneficial actions against stroke may be explained by the improved dilation of cerebral arterioles, cerebral blood flow, and remote (pre)conditioning in a rat model.^270^ Meta-analyses of CVOTs confirm the overall CV safety of GLP-1RA, with significant reductions in all-cause mortality.^271^ However, heterogeneity across trials, particularly in patient populations, baseline HbA1c levels, and concomitant CV medications, precludes generalization of the results across different populations.

The benefits of GLP-1RA are relatively clear for the reduction of atherosclerotic CV events in patients with diabetes or obesity. However, their impact on heart failure (HF) outcomes remains inconclusive, with evidence suggesting differing effects based on baseline cardiac function. Clinical trials evaluating the effects of GLP-1RA on HF outcomes present contrasting findings for patients with heart failure with reduced ejection fraction (HFrEF) versus those with preserved ejection fraction (HFpEF). In the FIGHT and LIVE trials, which collectively enrolled several hundred patients exclusively with HFrEF, treatment with liraglutide was associated with an increased incidence of HF-related adverse events.^272^, ^273^, ^274^ Similarly, in the EXSCEL trial, which included patients with a left ventricular ejection fraction (LVEF) ≤40%, exenatide was linked to an elevated risk of HF hospitalizations. A meta-analysis of FIGHT and EXSCEL data revealed that GLP-1RA use in patients with HFrEF significantly increased HF hospitalization risk (OR, 1.49; 95% CI, 1.05–2.10; *P* = .02).^274^, ^275^, ^276^ In contrast, much larger studies involving HFpEF patients suggest more favorable outcomes. In the STEP-HFpEF and STEP-HFpEF DM trials, semaglutide demonstrated improvements in health status and reductions in HF-related events, although these findings were based on a limited number of events.^277^^,^^278^ The SELECT trial, a landmark study, evaluated semaglutide in over 17,000 subjects with obesity and preexisting CVD, including 4286 patients with HF. This cohort consisted of 1341 with HFrEF, 2273 with HFpEF, and 666 with unclassified HF. Although semaglutide reduced CV events overall, its effects on specific HF subtypes remain unclear due to the lack of standardized HF characterization within the trial. Nevertheless, SELECT showed superiority of semaglutide over placebo for reducing CV events among people with obesity and preexisting CVD.^279^

Across the SELECT, FLOW, STEP-HFpEF, and STEP-HFpEF DM trials, a pooled analysis revealed that 3743 (16.8%) of 22,282 participants had a history of HFpEF. Among these, 1914 were randomized to semaglutide and 1829 to placebo. Semaglutide significantly reduced the risk of the combined endpoint of CV death or HF events, occurring in 5.4% of the semaglutide group compared with 7.5% in the placebo group (HR, 0.69; 95% CI, 0.53–0.89; *P* = .0045). The reduction in HF events was particularly noteworthy, with semaglutide showing a 41% risk reduction compared with placebo (2.8% vs 4.7%; HR, 0.59; 95% CI, 0.41–0.82; *P* = .0019). However, semaglutide did not demonstrate a significant effect on CV death alone (3.1% vs 3.7%; HR, 0.82; 95% CI, 0.57–1.16; *P* = .25). Importantly, fewer serious adverse events were reported in the semaglutide group compared with placebo (29.9% vs 38.7%), highlighting its favorable safety profile. These findings support the use of semaglutide as an effective therapy to reduce clinical heart failure events in HFpEF patients, a group for whom treatment options are currently limited.^280^ Therefore, GLP-1RA show differential effects on HF outcomes based on EF. Although they may benefit patients with HFpEF, evidence suggests caution in their use for HFrEF due to increased HF hospitalization risks. These findings underscore the need for more targeted trials to elucidate the role of GLP-1RA in HF management across different subtypes.

In conclusion, GLP-1RA offer robust CV benefits in T2DM, chronic kidney disease, and obesity, with evidence supporting their role as a preferred therapy for high-risk CV patients. Their role in HF is less clear and needs to be further investigated. Other indications for GLP-1RA therapy, such as Alzheimer and Parkinson disease, fatty liver disease, and peripheral artery disease (atherosclerosis), are still under investigation.^281^ The most important studies on cardioprotective effects of GLP-1R agonists are shown in Table 2.^282^ Cardiovascular actions and clinical outcomes with GLP-1RA are also reviewed in previous works.^108^^,^^282^^,^^283^ Whether GLP-1RA might be similarly useful in reducing rates of MACE in people without T2DM or obesity has not been fully explored; however, it would be worthwhile to investigate in detail in future studies.

**Table 2:** Major safety endpoints of selected GLP-1R agonists tested in CVOT GLP-1RA revealing a CV benefit relative to standard care are marked in bold. Reproduced from Helmstadter et al^134^ with permission.

Cardiovascular Outcome Trials of Selected GLP-1RAs						
Trial	ELIXA Lixisenatide	LEADER Liraglutide	SUSTAIN-6 Semaglutide	EXSCEL Exenatide	REWIND Dulaglutide	HARMONY Albiglutide
Primary outcome	1.02 (0.89–1.17) *P* < .001 for noninferiority *P* = .81 for superiority CV death, MI, stroke	**0.87 (0.78–0.97)** *P* < .001 for noninferiority ***P* = .01** for superiority CV death, MI, stroke	**0.74 (0.58–0.95)** *P* < .001 for noninferiority ***P* = .02** for superiority CV death, MI, stroke	0.91 (0.83–1.00) *P* < .001 for noninferiority *P* = .06 for superiority CV death, MI, stroke	**0.88 (0.79–0.99)** ***P* = .026** for superiority CV death, MI, stroke, UA	**0.78 (0.68–0.90)** *P* < .0001 for noninferiority ***P* = .0006** for superiority CV death, MI, stroke
Secondary outcome	1.0 (0.90–1.11) *P* = .96 CV death, MI, stroke, UA, HF hosp., revascul.	**0.88 (0.81–0.96)** ***P* = .005** CV death, MI, stroke, UA or HF hosp., revascul.	**0.74 (0.62–0.89)** ***P* = .002** CV death, MI, stroke, UA or HF hosp., revascul.	n/a	n/a	**0.78 (0.69–0.90)** ***P* = .0005** CV death, MI, stroke, urgent revascul. for UA, individual components of the primary endpoint, CV death/hospital admission because of HF
CV death	0.98 (0.78–1.22) *P* = .85	**0.78 (0.66–0.93)** ***P* = .007**	0.98 (0.65–1.48) *P* = .92	0.88 (0.76–1.02)	0.91 (0.78–1.06) *P* = .21	0.93 (0.73–1.19) *P* = .578
All-cause-death	0.94 (0.78–1.13) *P* = .5	**0.85 (0.74–0.97)** ***P* = .02**	1.05 (0.74–1.50) *P* = .79	0.86 (0.77–0.97)	**0.90 (0.80–1.01)** ***P* = .067**	0.95 (0.79–1.16) *P* = .644
HF hospitalization	0.96 (0.75–1.23) *P* = .75	0.87 (0.73–1.05) *P* = .14	1.11 (0.77–1.61) *P* = .57	0.94 (0.78–1.13)	0.93 (0.77–1.12)^a^ *P* = .46	0.85 (0.70–1.04)^b^ *P* = .113
Reduction of glycated hemoglobin	−0.27 pps (−0.31 to −0.22) *P* < .001	−0.40 pps (−0.45 to −0.34)	0.5 mg semaglutide: −0.7 pps 1 mg semaglutide: −1.0 pps	−0.53% (−0.57 to −0.50) *P* < .001	−0.61% (−0.65 to −0.58) *P* < .0001	difference at 8 months: −0.63% (−0.69 to −0.58) difference at 16 mo: −0.52% (−0.58 to −0.45)

CV death, cardiovascular death; HF, heart failure; hosp., hospitalization; MI, myocardial infarction; n/a, not available; pps, percentage points; revascul., revisualization; UA, unstable angina.

a Hospital admission for HF or urgent visit.

b Composite of death from cardiovascular causes or hospital admission for HF, ELIXA trial,^259^ LEADER trial,^217^ SUSTAIN-6 trial,^216^ EXSCEL trial,^276^ REWIND trial,^261^ and HARMONY trial.^284^

*Summary for the clinical practice*: DPP4i, GLP-1RA, and SGLT2i are considered safe options for managing T2DM with co-existing CVD. Although all 3 drug classes ensure CV safety, only GLP-1RA and SGLT2i demonstrate cardioprotective effects beyond glycemic control. Notably, GLP-1RA and SGLT2i have shown significant benefits in patients with overweight/obesity, atherosclerosis, or chronic kidney disease, even in the absence of T2DM. These agents have been associated with marked improvements in CV endpoints, such as reduced hospitalizations for HF and CV mortality. These findings underscore the unique role of GLP-1RA and SGLT2i as preferred therapeutic options for patients with elevated CV risk. By offering benefits that extend beyond glycemic management, these drug classes have become integral to the modern treatment paradigm for T2DM and associated CV conditions.

### B Cardiovascular safety of dipeptidyl peptidase 4 inhibitors: evidence from major trials

Data from CVOTs, including TECOS, EXAMINE, SAVOR-TIMI53, CARMELINA, and CAROLINA, demonstrate the CV safety of DPP-4i in patients with T2DM. These trials demonstrated noninferiority of DPP-4i compared with placebo or active comparators, such as glimepiride, for primary CV endpoints. However, unlike GLP-1RA or SGLT-2i, DPP-4i did not reduce MACE.

The SAVOR-TIMI 53 trial highlighted a potential safety concern with saxagliptin. Although saxagliptin demonstrated noninferiority for MACE, it was associated with a 27% increased risk of hospitalization for HF (HR, 1.27; 95% CI, 1.07–1.51).^285^ This finding has raised concerns about saxagliptin use in patients with pre-existing HF or those at high risk for HF. Regulatory agencies, including the Food and Drug Administration (FDA), have since added warnings to saxagliptin’s prescribing information to caution against its use in such populations. The CAROLINA trial further explored the CV safety of linagliptin compared with glimepiride. Although linagliptin did not improve CV outcomes, it provided better glycemic control, with more patients maintaining HbA1c levels below 7.0% (16.0% vs 10.2%) and significantly fewer hypoglycemic events (10.6% vs 37.7%).^286^

Mechanistically, DPP-4i reduce atherosclerosis and inflammation in preclinical models.^92^^,^^105^^,^^120^ For example, sitagliptin reduced coronary plaque volume and non-HDL cholesterol levels, whereas linagliptin decreased aortic pulse wave velocity, a surrogate marker for arterial stiffness in animal models.^92^^,^^94^^,^^287^ Notably, the evidence supporting ant-inflammatory effects of DPP4i in patients with T2DM and CVD is much less compelling.^128^ Despite some promising preclinical findings, the adverse HF outcomes seen in saxagliptin trials underscore the need for caution when prescribing this drug to high-risk CV populations.^288^ Nevertheless, a meta-analysis^289^ and a large Canadian cohort study^290^ did not support generalization of this side effect to other DPP-4i. Although DPP-4i remain widely used for their tolerability, overall safety, and low risk of hypoglycemia, their limited efficacy in reducing CV events and concerns about HF risk position them behind GLP-1RA and SGLT-2i in current guidelines.^291^

*Summary for the clinical practice*: DPP-4i provide a useful option for glycemic control in T2DM but are best reserved for patients without elevated CV or HF risks. Cardiovascular actions and clinical outcomes with DPP-4i are also reviewed in a previous work.^283^

### C Cardiovascular outcomes with sodium-glucose cotransporter 2 inhibitors: evidence from major trials

**Fig. 12:**
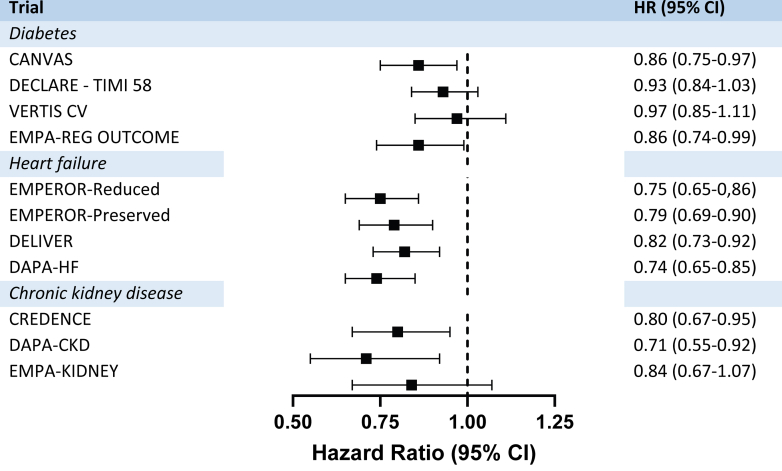
The overall effect of SGLT2 inhibition on the MACE composite. The overview figure was composed de novo from the single study results. Most studies report the outcome for cardiovascular deaths and hospitalizations due to HF as the primary endpoint. Some studies also include stroke and myocardial infarction in their results.^218^^,^^219^^,^^293^^,^^294^ CANVAS,^293^ DECLARE—TIMI 58,^218^ VERTIS CV,^295^ EMPA-REG OUTCOME,^219^ EMPEROR-Reduced,^296^ EMPEROR-Preserved,^297^ DELIVER,^254^ DAPA-HF,^298^ CREDENCE,^294^ DAPA-CKD,^255^ and EMPA-KIDNEY.^299^ For a complete picture we refer to the meta-analysis data published previously.^292^

Recently, a SGLT2 Inhibitor Meta-Analysis Cardio-Renal Trialists' Consortium collaborative meta-analysis revealed SGLT2i to be associated with a 9% reduction in MACE, driven primarily by reductions in CV deaths, particularly from HF and sudden cardiac death (Fig. 12).^292^, ^293^, ^294^, ^295^, ^296^, ^297^, ^298^, ^299^ These benefits are consistent across subgroups, including those with or without atherosclerotic CVD, diabetes, HF, and across a broad range of kidney functions. Remarkably, the efficacy persisted even in patients with an eGFR <30 mL/min/1.73 m^2^, a subgroup where diminished urinary glucose excretion had raised concerns about efficacy.^292^

The CV benefits of SGLT2i have been robustly demonstrated in several large trials. EMPA-REG OUTCOME (empagliflozin) showed a 38% reduction in CV mortality and a 35% reduction in HF hospitalizations among patients with T2DM and established atherosclerotic CVD.^219^ CANVAS (canagliflozin) demonstrated a 14% reduction in MACE but reported an increased risk of amputations.^293^ DECLARE-TIMI 58 (dapagliflozin) highlighted a 27% reduction in HF hospitalizations, with neutral effects on MACE in a broader population, including those without atherosclerotic CVD.^218^ In HFrEF, DAPA-HF (dapagliflozin)^298^ and EMPEROR-Preserved (empagliflozin)^297^ reported reductions in combined CV mortality and HF hospitalizations by 26% and 25%, respectively.

HFpEF has also been studied, with EMPEROR-Preserved (empagliflozin) demonstrating a 21% reduction in the combined endpoint of heart failure hospitalizations and CV death.^297^ SOLOIST-WHF (sotagliflozin), focused on patients recently hospitalized for HF, showed significant reductions in HF-related events.^300^ VERTIS CV (ertugliflozin) confirmed CV safety but did not demonstrate substantial decreases in MACE or heart failure outcomes.^301^

*Summary for the clinical practice:* These findings highlight the multifaceted benefits of SGLT2i, particularly in reducing heart failure-related risks and CV mortality. The relatively modest effect on MI and the absence of stroke reduction align with the primary mechanisms of SGLT2i, which focus on HF and kidney protection rather than direct antiatherosclerotic actions. The heterogeneity in CV outcomes across these trials underscores the importance of patient selection. Trials like EMPA-REG OUTCOM and CANVAS, targeting high-risk atherosclerotic CVD populations, demonstrated more pronounced MACE reductions. In contrast, broader studies like DECLARE-TIMI 58 revealed neutral effects in patients without atherosclerotic CVD. In conclusion, SGLT2i offer consistent CV benefits, particularly in reducing HF hospitalizations and CV mortality across a broad spectrum of patients. How this is achieved mechanistically is not clear, as SGLT-2 is not expressed in the heart, and some preclinical studies link SGLT2i-mediated cardioprotection to mechanisms independent of SGLT-2.^302^

### D Combination therapy with glucagon-like peptide 1 receptor agonists and sodium-glucose cotransporter 2 inhibitors in type 2 diabetes mellitus: cardiovascular outcomes and emerging evidence

The management of T2DM with atherosclerotic CVD increasingly prioritizes CV risk reduction. Although current guidelines advocate the use of GLP-1RA or SGLT2i for these patients, the benefit of combining these agents is under investigation.^291^ As described above, DPP4i have shown CV neutrality in trials and limited efficacy when combined with SGLT2i or GLP-1RA, thus reducing their role in comprehensive T2DM management. The focus has shifted toward GLP-1RA and SGLT2i combinations due to their complementary mechanisms and potential additive CV benefits. Meta-analyses of the Harmony Outcomes and AMPLITUDE-O trials, the largest data set of patients on baseline SGLT2i, highlight the CV effects of adding GLP-1RA.^303^ In the analysis of Harmony Outcomes, albiglutide significantly reduced MACE—CV death, MI, or stroke—as well as HF hospitalization, regardless of background SGLT2i use.^303^ Similarly, AMPLITUDE-O confirmed that efpeglenatide provided MACE reductions independently of SGLT2i use.^304^

Although direct evidence of additive CV benefits remains limited, trials such as DURATION-8 have demonstrated additive effects on metabolic outcomes. Combining GLP-1RA and SGLT2i leads to greater HbA1c, weight, and blood pressure reductions than monotherapy.^305^ Also, the cumulative incidence of MACE and serious renal events was significantly decreased by 30% and 57%, respectively, by the combination therapy with GLP-1RA and SGLT2i compared with GLP-1RA monotherapy.^306^ In a comparison of the combination therapy with GLP-1RA and SGLT2i to SGLT2i monotherapy, MACE incidence was still decreased by 29%. These benefits align with their distinct mechanisms: GLP-1RA improve glycemic control, lipid profiles, and atherosclerosis, whereas SGLT2i enhance renal and hemodynamic functions, reduce inflammation, and improve cardiac metabolism. Also, markers of oxidative stress in T2DM patients were more efficiently decreased, and vascular markers (eg, glycocalyx)/effective cardiac work were ameliorated more pronounced by the combination therapy with GLP-1RA and SGLT2i than by monotherapy with these drugs or insulin alone.^307^^,^^308^ Notably, the results of the SOUL trial studying the CV safety of oral semaglutide in people with T2DM demonstrated a 14% reduction in the primary composite outcome, with 49% of trial participants taking a SGLT2i at some point during the trial.^309^

*Summary for the clinical practice:* Safety for combination therapy remains a key consideration. Harmony Outcomes trial data confirmed that adding GLP-1RA to SGLT2i did not increase the risk of adverse events, including hypoglycemia or diabetic retinopathy.^303^ Nevertheless, current guidelines do not routinely recommend combining GLP-1RA and SGLT2i due to limited evidence from randomized trials, cost considerations, and the lack of clear CV superiority over monotherapy.^291^ The relatively small proportion of patients on SGLT2i in prior trials further limits the generalizability of findings. In conclusion, GLP-1RA and SGLT2i independently provide substantial CV benefits in T2DM with atherosclerotic CVD. Preliminary evidence suggests their combination is safe and may offer additional benefits, but robust randomized trials are needed to confirm these effects and refine treatment recommendations.

### E The role of glucagon-like peptide 1 receptor agonists in weight management for nondiabetic overweight and obese adults

The global obesity epidemic presents a significant health and economic burden, with a growing prevalence of elevated body mass index (BMI). In the United States, 70% of adults are classified as overweight or obese, and projections indicate that nearly half of the population may be obese by 2030. Overweight individuals are defined by a BMI ≥ 25 kg/m^2^, whereas obesity is defined as a BMI ≥ 30 kg/m^2^.^310^ Obesity is a major independent risk factor for T2DM due to its association with insulin resistance and metabolic abnormalities, including hyperinsulinemia, hyperglycemia, and dyslipidemia.^311^ Excess adipose tissue contributes to insulin resistance by releasing nonesterified fatty acids, tumor necrosis factors, and proinflammatory cytokines.^312^ With each kilogram of weight gained, diabetes risk increases by 4.5%–9%, and individuals with a BMI > 30 have a 20-fold higher risk of developing T2DM. Weight loss improves glucose homeostasis and reduces cardiometabolic risk in diabetic patients.^313^ For overweight and obese individuals without T2DM, weight reduction is also useful for preventing the onset of diabetes. Current pharmacological options for obesity management include lipase inhibitors (orlistat), opioid receptor antagonists (naltrexone/bupropion), and adrenergic agonists (phentermine/topiramate).^314^ Additionally, GLP-1RA, such as tirzepatide, liraglutide, and semaglutide, originally approved for glycemic control, have gained approval as effective weight loss agents.^315^^,^^316^

GLP-1 delays gastric emptying and promotes satiety, collectively contributing to weight loss. Since the approval of liraglutide (Saxenda, 3 mg) for weight management in nondiabetic adults in 2014, GLP-1RA have transformed obesity treatment. Semaglutide is approved at a maximum dose of 2.4 mg for weight loss therapy and has shown superior efficacy to previously approved classes of weight loss agents. Although most antiobesity drugs achieve less than 10% weight reduction at tolerable doses, semaglutide demonstrated a 14.9% reduction in body weight in clinical trials.^315^^,^^316^

**Fig. 13:**
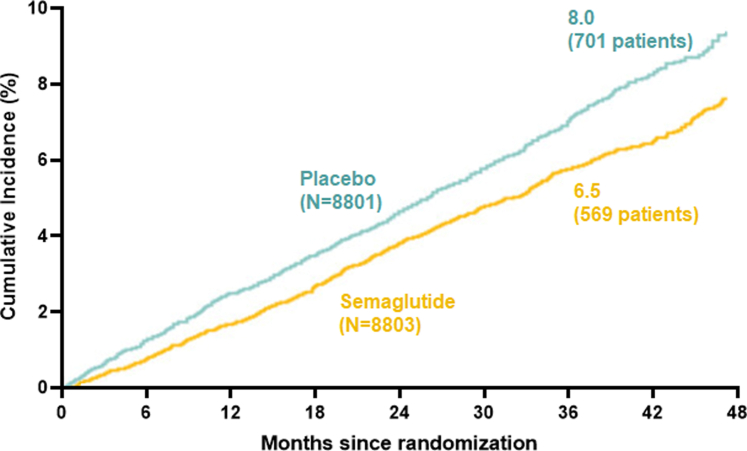
Time-to-first-event analysis for primary and confirmatory secondary efficacy end points for GLP-1RA therapy of CVD patients with obesity but without diabetes. The graph shows the cumulative incidence of the first affirmative secondary endpoint (death from cardiovascular causes). Overall HR for death from cardiovascular causes (nonfatal MI or stroke) for the semaglutide intervention was 0.8 (95% CI: 0.72–0.9) with *P* < .001 for superiority. Reproduced from Lincoff et al^279^ with permission.

In 2021, the FDA approved semaglutide 2.4 mg for chronic weight management in obese adults or those overweight with comorbidities to be combined with caloric restriction and increased physical activity. Semaglutide is available as a once-weekly subcutaneous injection or a daily oral formulation. For T2DM, beyond weight loss, GLP-1RA have been shown to reduce the rates of MACE in separate trials for T2DM or overweight/obesity. The SELECT trial studied 17,604 nondiabetic individuals with obesity or overweight (BMI ≥ 27) and pre-existing CVD, revealing a 9.39% mean weight reduction over 104 weeks with semaglutide. Semaglutide reduced the composite primary endpoint of CV death, nonfatal MI, or stroke by 20% compared with placebo (HR, 0.80; 95% CI, 0.72–0.90; *P* < .001) over a mean follow-up of more than 3 years (Fig. 13). Importantly, all-cause mortality was reduced by 19%. Adverse events led to higher discontinuation in the semaglutide group (16.6% vs 8.2%). These findings establish semaglutide as an effective CV risk reduction therapy in overweight and obese patients without diabetes.^279^

*Summary for the clinical practice:* This growing evidence base highlights the potential of GLP-1 RAs, particularly semaglutide, as powerful agents in weight management and as a promising approach for mitigating CV risks associated with obesity in nondiabetic populations.

### F Sodium-glucose cotransporter 2 inhibitors in nondiabetic heart failure

Cardiovascular complications associated with T2DM include coronary artery disease, HF, atrial fibrillation, ischemic strokes, and peripheral arterial disease. Furthermore, diabetes heightens the likelihood of albuminuria and chronic kidney disease, both of which are independent risk factors for CVD. HF is currently classified based on EF: HF with reduced EF (HFrEF; EF ≤ 40%), HF with preserved EF (HFpEF; EF > 50%), and HF with mildly reduced EF (HFmrEF; EF 40%-50%).^317^^,^^318^ The interplay between diabetes and cardiorenal comorbidities leads to a cumulative increase in the risk of CV events and mortality. Although the glucose-lowering class of drugs like SGLT2i can reduce CV consequences driven by T2DM, recent studies demonstrated SGLT2i to be effective in HF patients without T2DM.

*Summary for the clinical practice:* The use of SGLT2i in HF has dramatically transformed patient outcomes. Initially developed for glycemic control, their role in HF management gained recognition after the EMPA-REG OUTCOME trial demonstrated a significant reduction in HF hospitalizations in the empagliflozin group compared with placebo. These results suggested that SGLT2i directly affect HF, independent of their glucose-lowering properties.

#### 1 Sodium-glucose cotransporter 2 inhibitors in heart failure with reduced ejection fraction

HFrEF, a LVEF of ≤ 40%, shows significant improvements with SGLT2i. The DAPA-HF trial demonstrated that dapagliflozin reduced primary CV outcomes—including CV death, hospitalization for HF, or urgent hospital visits requiring intravenous HF therapy—by 26% in patients with EF ≤ 40% and an eGFR ≥ 30 mL/min/1.73 m^2^. Similarly, the EMPEROR-Reduced trial evaluated empagliflozin in patients with a mean EF of 27% and an eGFR ≥ 20 mL/min/1.73 m^2^, reporting a 25% reduction in the combined endpoints of CV death and HF hospitalization. In addition, a slower decline in kidney function was observed, highlighting the dual benefits of SGLT2i in managing HFrEF. These findings have established SGLT2i as a cornerstone and part of the guidelines in HFrEF treatment, significantly improving morbidity and mortality outcomes.^317^^,^^318^

#### 2 Sodium-glucose cotransporter 2 inhibitors in heart failure with preserved ejection fraction and heart failure with mildly reduced ejection fraction

HF has historically been challenging to treat. The EMPEROR-Preserved trial demonstrated that empagliflozin improved CV outcomes in HFpEF patients, achieving a 21% reduction in the combined endpoint of CV death and HF hospitalization. This landmark trial included HFmrEF and HFpEF patients, irrespective of diabetes status.^297^ The DELIVER trial further confirmed these results with dapagliflozin, showing an 18% reduction in the primary composite endpoint of HF worsening or CV death in patients with HFmrEF and HFpEF.^254^

#### 3 Sodium-glucose cotransporter 2 inhibitors in acute heart failure

Acute heart failure is a critical clinical condition requiring immediate intervention. The SOLOIST-WHF trial investigated sotagliflozin in patients recently hospitalized for worsening HF and found a significant reduction in the combined endpoint of CV death, HF hospitalization, or urgent HF visits.^300^ Meta-analyses have further demonstrated that SGLT2i improve Kansas City Cardiomyopathy Questionnaire scores and reduce the risk of HF rehospitalization without increasing the risk of hypotension or acute kidney injury. However, no significant effect on all-cause mortality was observed.^254^ Although improvements in LVEF were primarily observed in HFrEF patients at advanced stages of HF, SGLT2i have shown broad clinical utility in acute and chronic HF settings, significantly enhancing treatment outcomes.

### G Modern antidiabetic drugs also for the treatment of hypertension?

The antihypertensive properties of GLP-1RA, DPP-4i, and SGLT2i are clinically meaningful benefits beyond glycemic control. These agents reduce blood pressure (BP) via different mechanisms including natriuresis, weight loss, endothelial modulation, and autonomic regulation.^319^, ^320^, ^321^ Clinical trials and animal studies over the past 15 years have detailed the magnitude of these effects, which vary by drug class.

GLP-1RA such as liraglutide and semaglutide consistently reduce systolic BP by approximately 2–5 mmHg in meta-analyses^321^ and a high-quality cohort study.^279^ Although weight loss contributes to this effect, GLP-1 receptor activation also promotes natriuresis through direct renal tubular actions and increased atrial natriuretic peptide (ANP) secretion, resulting in decreased aldosterone and plasma volume.^321^^,^^322^ Additionally, GLP-1RA improve endothelial nitric oxide bioavailability and attenuate oxidative stress, promoting vasodilation in experimental studies.^120^^,^^141^^,^^323^ Experimental data also support improved vascular reactivity and reduced inflammation in hypertensive mice under GLP-1RA treatment.^129^ There is also evidence of central sympathetic modulation, although the mild increase in heart rate observed remains unclear.^324^

DPP-4 inhibitors (eg, sitagliptin, saxagliptin, and vildagliptin) exhibit mild and inconsistent BP-lowering effects, with small studies showing modest systolic BP reductions and larger trials failing to confirm them.^325^, ^326^, ^327^ These agents preserve vasoactive peptides such as GLP-1, brain natriuretic peptide, and substance P, which enhance vasodilation. They also exert endothelial anti-inflammatory and antioxidative effects.^120^^,^^328^ However, the absence of an effect on sodium excretion or weight impact limits their clinical BP effects, consistent with neutral CV outcomes in large trials.^285^^,^^327^

SGLT2 inhibitors (eg, empagliflozin, dapagliflozin, and canagliflozin) demonstrate robust BP reductions, typically 3–5 mmHg systolic, across multiple trials.^218^^,^^219^ By inhibiting sodium and glucose reabsorption in the proximal tubule, they induce natriuresis and osmotic diuresis, reducing intravascular volume and afterload.^329^^,^^330^ Additional salutary effects include improved arterial compliance, reduced sympathetic activity, and beneficial vascular and renal changes.^329^^,^^330^ Hypertensive rat models treated chronically with SGLT2i exhibit attenuated BP progression and reduced heart rate. Notably, BP effects appear early and are sustained without reflex tachycardia.^331^

These antihypertensive effects likely contribute to CV benefits observed in major CVOTs. In LEADER and SUSTAIN-6 (GLP-1RA)^216^^,^^217^ and EMPA-REG OUTCOME and DECLARE-TIMI 58 (SGLT2i),^218^^,^^219^ reductions in MACE, HF hospitalizations, and renal progression were documented, with treatment arms displaying consistently lower BP by several mmHg, as also reviewed in detail.^326^ BP lowering plausibly mediates a portion of the risk reduction, particularly for HF. In contrast, DPP-4i showed neither BP reduction nor CV benefit.^285^^,^^327^ Therefore, although BP reduction does not fully explain the cardioprotection provided by GLP-1RA and SGLT2i—given their multifactorial metabolic and organ-protective effects—it constitutes a significant therapeutic mediator and should be a focus of future mediation analyses.

## V Unwanted side effects of modern therapy for type 2 diabetes mellitus

DPP-4 inhibitors are generally well-tolerated, with a low incidence of adverse effects and minimal risk of hypoglycemia when used alone. The most common side effects are mild, such as upper respiratory tract infections, nasopharyngitis, headaches, urinary tract infections, and arthralgias.^332^ However, several infrequent but serious adverse reactions have been identified. Postmarketing surveillance has linked DPP-4i to severe, disabling joint pain, prompting the FDA to add class-wide label warnings in 2015.^333^ Rare cases of bullous pemphigoid have also been associated with DPP-4i; a meta-analysis of randomized trials found a significantly elevated risk with this class.^334^ In CVOTs, DPP-4i showed overall neutral effects on atherothrombotic events, but 1 trial (SAVOR-TIMI 53) noted a higher rate of hospitalization for HF with saxagliptin (3.5% vs 2.8% over ∼2 years, HR, 1.27).^285^ This finding led regulators to warn about potential HF risk with saxagliptin and alogliptin.^335^ Incretin-based therapies were once suspected to increase pancreatitis risk, but large trials have not confirmed a significant difference in pancreatitis incidence between DPP-4i and placebo.^285^ Notably, adjudicated rates of acute pancreatitis in SAVOR were low (≈0.3%) and virtually identical to placebo.^285^

GLP-1RA commonly cause gastrointestinal adverse effects. Nausea, vomiting, and diarrhea are frequently reported and can lead to treatment discontinuation.^217^ In the LEADER trial of liraglutide, for example, gastrointestinal symptoms were the most common adverse events prompting drug discontinuation.^217^ Such effects are dose-dependent and often diminish over time, but in some patients, they limit tolerability. GLP-1RA have also been associated with gallbladder disease: a post hoc analysis of LEADER found a higher incidence of acute gallbladder or biliary events in the liraglutide group than in the placebo group (141 of 4668 vs 88 of 4672 patients; HR, 1.60, *P* < .001).^216^ Regarding pancreatic safety, initial reports raised alarms about pancreatitis (and even pancreatic cancer) with incretin therapies. However, subsequent high-quality evidence has not substantiated a meaningful increase in pancreatitis risk.^217^ In LEADER, for instance, the incidence of acute pancreatitis was low and not significantly different from placebo.^217^ Nonetheless, product labels advise caution in patients with a history of pancreatitis. An unexpected finding in the semaglutide CV outcomes trial (SUSTAIN-6) was an elevated rate of diabetic retinopathy complications (3.0% with semaglutide vs 1.8% with placebo over ∼2 years).^336^ This has been attributed to the rapid glycemic improvement with semaglutide and underscores the need for careful retinal monitoring in patients with preexisting retinopathy. Finally, although rodent studies linked GLP-1RA to C-cell tumors, large human studies have found no increase in thyroid cancer among GLP-1RA-treated patients (pooled HR, ∼0.8 vs DPP-4 inhibitor comparators).^337^ Accordingly, the class carries a precaution against use in patients with a personal or family history of medullary thyroid carcinoma, but current evidence has been reassuring regarding thyroid safety in the general population.^337^ These and other adverse effects of GLP-1RA therapy (eg, antibody formation against the drugs) were previously summarized by previous work.^246^^,^^281^

SGLT2 inhibitors promote glycosuria and have side effects related to urinary glucose loss. Genital mycotic infections are among the most common adverse effects, occurring at significantly higher frequency than in placebo groups. Urinary tract infections are also more frequent; although most are mild, rare cases of urosepsis and pyelonephritis have been reported, leading the FDA to add warnings about serious urinary infections with this class.^338^ By causing osmotic diuresis, SGLT2i can precipitate volume depletion, hypotension, and electrolyte imbalances in susceptible patients. Another important risk is diabetic ketoacidosis: SGLT2i have been linked to euglycemic or minimally hyperglycemic diabetic ketoacidosis, with dozens of cases reported in the first few years of clinical use. Regulatory agencies mandated label warnings about ketoacidosis in 2015, and prescribers are advised to monitor for symptoms of metabolic acidosis.^338^ Moreover, in 2018, the FDA warned of rare cases of necrotizing fasciitis of the perineum (Fournier’s gangrene) in patients on SGLT2i.^339^ By early 2019, an FDA review had identified 55 cases of Fournier’s gangrene in patients receiving SGLT2i (male and female).^340^ This led to a new class warning, and clinicians should remain vigilant for this life-threatening infection. Lower urinary tract symptoms of SGLT2i are also reviewed in previous works.^341^ Finally, 1 agent—canagliflozin—was associated with an approximately 2-fold increase in lower-limb amputation risk in 1 large CV outcome trial (CANVAS).^293^ This amputation signal was specific to canagliflozin and was not observed with other SGLT2i in similar trials. Canagliflozin’s label briefly carried a boxed warning for amputations, which was later removed after further data, but this risk is still considered in patient selection and monitoring.

## VI Clinical outlook and conclusions

Endothelial dysfunction is a common and early symptom of CVD,^342^ particularly coronary heart disease, and is linked to future CV events in CVD patients.^343^ Oxidative stress, a prognostic marker, is also associated with CVD in humans^61^^,^^344^^,^^345^ and is a potential target for therapy.^346^ Recent data suggest a close correlation between oxidative stress and inflammation in the vasculature (especially under diabetic conditions),^35^^,^^347^ making both independent risk factors for CVD progression in humans.^64^^,^^69^ Using compounds for the inhibition of specific ROS sources and cell-specific scavenging of ROS or activators of intrinsic antioxidant systems, such as NRF2-dependent pathways, is a promising strategy.^348^ Also, exploring the pleiotropic antioxidant and anti-inflammatory properties of established CV drugs seems reasonable.^74^ Screening for potent anti-inflammatory effects and prescreening patients for inflammation and oxidative stress markers could help develop personalized medical treatments.

The antioxidant and anti-inflammatory properties of the modern antidiabetic drugs GLP-1RA and SGLT2i, along with other pleiotropic health effects besides their principal mode of action, preventing glucotoxicity, provide a reasonable explanation for their highly beneficial impact on diabetic complications and associated comorbidities—important prognostic endpoints that are less affected by DPP-4i therapy. The promising results with GLP-1RA and DPP-4 inhibition in endotoxemia and atherosclerosis models support their potent anti-inflammatory effects. Also, SGLT2i displayed potent anti-inflammatory effects in T1DM and T2DM models. Common to all these compounds were the antioxidant effects and efficient prevention of oxidative damage in various tissues. In summary, GLP-1RA and SGLT2i are primarily safe, well-proven, and highly effective drugs to prevent CV complications and renal impairment in T2DM patients at medium to high risk. GLP-1RA and SGLT2i, despite being developed initially as antidiabetic medications, have demonstrated significant CV benefits even in patients without diabetes. GLP-1RA appear to reduce CV events in humans primarily through their anti-inflammatory properties, likely complemented by the additional benefits of reduced BP and body weight, which improve metabolic profiles and reduce CV risk factors.^152^^,^^279^^,^^312^ Immunomodulation seems to be a key mechanism of the pleiotropic cardioprotective effects of GLP-1- and SGLT2-based drugs, also supported by the promising results of the large clinical trials on monoclonal antibodies against IL-1*β* in patients with T2DM.^349^^,^^350^

On the other hand, SGLT2i, with their diuretic-like mechanisms, are particularly advantageous in patients with HF across the spectrum of EF (HFrEF, HFmrEF, and HFpEF), as well as those with cardiorenal syndromes.^254^^,^^279^^,^^297^ Both drug classes have already secured a prominent role in managing CVDs, and their clinical utility is expected to expand further in the coming years as more evidence emerges.^291^^,^^318^

Evidence is growing that GLP-1RA may represent a therapeutic strategy against Alzheimer and Parkinson disease, fatty liver disease, and peripheral artery disease (atherosclerosis)^281^ as well as hypertension (see above) beyond glycemic control and weight loss. Also, SGLT2i may be used for new indications such as fatty liver disease by reducing hepatic, perivisceral, pericardial, and perivascular fat accumulation, thereby attenuating obesity-related inflammation.^351^ SGLT2 inhibitors also display a systemic, BP-lowering effect without generating a reflex chronotropic response, prevent vascular aging, and improve energy metabolism from ketone bodies and lipid consumption, thereby optimizing energy efficiency in the myocardium. These pleiotropic effects will open attractive new markets and health benefits for a much broader target group.

## Conflicts of interest

Andreas Daiber reports financial support provided by Mainz Heart Foundation. Thomas Munzel reports financial support provided by German Center for Cardiovascular Disease. Sebastian Steven reports financial support provided by Else Kroner-Fresenius Foundation. Andreas Daiber and Thomas Münzel received research grant support from Boehringer Ingelheim Pharma GmbH & Co KG, Ingelheim (more than 5 years ago).

## References

[1] Quianzon C.C., Cheikh I. (2012). History of insulin. J Community Hosp Intern Med Perspect.

[2] Ramachandra Bhat L., Vedantham S., Krishnan U.M., Rayappan J.B.B. (2019). Methylglyoxal—an emerging biomarker for diabetes mellitus diagnosis and its detection methods. Biosens Bioelectron.

[3] Leslie R.D., Evans-Molina C., Freund-Brown J. (2021). Adult-onset type 1 diabetes: current understanding and challenges. Diabetes Care.

[4] American Diabetes Association Professional Practice Committee (2024). 2. Diagnosis and classification of diabetes: standards of care in diabetes-2024. Diabetes Care.

[5] Injury I., Prevalence C., Disease GBD (2018). Global, regional, and national incidence, prevalence, and years lived with disability for 354 diseases and injuries for 195 countries and territories, 1990-2017: a systematic analysis for the Global Burden of Disease Study 2017. Lancet.

[6] (2021). International Diabetes Federation, IDF Diabetes Atlas.

[7] Lim S.S., Vos T., Flaxman A.D. (2012). A comparative risk assessment of burden of disease and injury attributable to 67 risk factors and risk factor clusters in 21 regions, 1990-2010: a systematic analysis for the Global Burden of Disease Study 2010. Lancet.

[8] Murray C.J., Ezzati M., Flaxman A.D. (2012). GBD 2010: design, definitions, and metrics. Lancet.

[9] Nakagami T., Qiao Q., Tuomilehto J. (2006). Screen-detected diabetes, hypertension and hypercholesterolemia as predictors of cardiovascular mortality in five populations of Asian origin: the DECODA study. Eur J Cardiovasc Prev Rehabil.

[10] Gerstein H.C., Swedberg K., Carlsson J. (2008). The hemoglobin A1c level as a progressive risk factor for cardiovascular death, hospitalization for heart failure, or death in patients with chronic heart failure: an analysis of the Candesartan in Heart failure: Assessment of Reduction in Mortality and Morbidity (CHARM) program. Arch Intern Med.

[11] Bozkurt B., Aguilar D., Deswal A. (2016). Contributory risk and management of comorbidities of hypertension, obesity, diabetes mellitus, hyperlipidemia, and metabolic syndrome in chronic heart failure: a scientific statement from the american heart association. Circulation.

[12] Ghoneim S., Dhorepatil A., Shah A.R. (2020). Non-alcoholic steatohepatitis and the risk of myocardial infarction: A population-based national study. World J Hepatol.

[13] Ferdinandy P., Andreadou I., Baxter G.F. (2023). Interaction of cardiovascular nonmodifiable risk factors, comorbidities and comedications with ischemia/reperfusion injury and cardioprotection by pharmacological treatments and ischemic conditioning. Pharmacol Rev.

[14] Shrestha S.S., Honeycutt A.A., Yang W. (2018). Economic costs attributable to diabetes in each U.S. state. Diabetes Care.

[15] Health Effects Institute (2024). State of Global Air 2024: Special Report. https://www.stateofglobalair.org/resources/report/state-global-air-report-2024.

[16] Global Burden of Disease Risk Factors Collaborators (2020). Global burden of 87 risk factors in 204 countries and territories, 1990-2019: a systematic analysis for the Global Burden of Disease Study 2019. Lancet.

[17] UK Prospective Diabetes Study (UKPDS) Group (1998). Intensive blood-glucose control with sulphonylureas or insulin compared with conventional treatment and risk of complications in patients with type 2 diabetes (UKPDS 33). UK Prospective Diabetes Study (UKPDS) Group. Lancet.

[18] Holman R.R., Paul S.K., Bethel M.A., Matthews D.R., Neil H.A. (2008). 10-year follow-up of intensive glucose control in type 2 diabetes. N Engl J Med.

[19] Holt R.I.G., DeVries J.H., Hess-Fischl A. (2021). The management of type 1 diabetes in adults. A consensus report by the American Diabetes Association (ADA) and the European Association for the Study of Diabetes (EASD). Diabetologia.

[20] Martin-Montalvo A., Mercken E.M., Mitchell S.J. (2013). Metformin improves healthspan and lifespan in mice. Nat Commun.

[21] Jansen T., Kvandova M., Daiber A. (2020). The AMP-activated protein kinase plays a role in antioxidant defense and regulation of vascular inflammation. Antioxidants.

[22] Hardie D.G. (2014). AMPK--sensing energy while talking to other signaling pathways. Cell Metabol.

[23] Fisman E.Z., Tenenbaum A. (2009). A cardiologic approach to non-insulin antidiabetic pharmacotherapy in patients with heart disease. Cardiovasc Diabetol.

[24] Han Y., Xie H., Liu Y., Gao P., Yang X., Shen Z. (2019). Effect of metformin on all-cause and cardiovascular mortality in patients with coronary artery diseases: a systematic review and an updated meta-analysis. Cardiovasc Diabetol.

[25] Wang F., He Y., Zhang R., Zeng Q., Zhao X. (2017). Combination therapy of metformin plus dipeptidyl peptidase-4 inhibitor versus metformin plus sulfonylurea and their association with a decreased risk of cardiovascular disease in type 2 diabetes mellitus patients. Medicine.

[26] Bain S., Druyts E., Balijepalli C. (2017). Cardiovascular events and all-cause mortality associated with sulphonylureas compared with other antihyperglycaemic drugs: a Bayesian meta-analysis of survival data. Diabetes Obes Metab.

[27] Cheng D., Gao H., Li W. (2018). Long-term risk of rosiglitazone on cardiovascular events—a systematic review and meta-analysis. Endokrynol Pol.

[28] Zhang Z., Chen X., Lu P. (2017). Incretin-based agents in type 2 diabetic patients at cardiovascular risk: compare the effect of GLP-1 agonists and DPP-4 inhibitors on cardiovascular and pancreatic outcomes. Cardiovasc Diabetol.

[29] Liu D., Jin B., Chen W., Yun P. (2019). Dipeptidyl peptidase 4 (DPP-4) inhibitors and cardiovascular outcomes in patients with type 2 diabetes mellitus (T2DM): a systematic review and meta-analysis. BMC Pharmacol Toxicol.

[30] Toyama T., Neuen B.L., Jun M. (2019). Effect of SGLT2 inhibitors on cardiovascular, renal and safety outcomes in patients with type 2 diabetes mellitus and chronic kidney disease: a systematic review and meta-analysis. Diabetes Obes Metabol.

[31] Steven S., Frenis K., Oelze M. (2019). Vascular inflammation and oxidative stress: major triggers for cardiovascular disease. Oxid Med Cell Longev.

[32] Steven S., Frenis K., Oelze M., Preedy V. (2020). Diabetes—Oxidative Stress and Dietary Antioxidants.

[33] Daiber A., Steven S., Weber A. (2017). Targeting vascular (endothelial) dysfunction. Br J Pharmacol.

[34] Daiber A., Di Lisa F., Oelze M. (2017). Crosstalk of mitochondria with NADPH oxidase via reactive oxygen and nitrogen species signalling and its role for vascular function. Br J Pharmacol.

[35] Wenzel P., Kossmann S., Munzel T., Daiber A. (2017). Redox regulation of cardiovascular inflammation—Immunomodulatory function of mitochondrial and Nox-derived reactive oxygen and nitrogen species. Free Radic Biol Med.

[36] Daiber A., Steven S., Vujacic-Mirski K. (2020). Regulation of vascular function and inflammation via cross talk of reactive oxygen and nitrogen species from mitochondria or NADPH oxidase-implications for diabetes progression. Int J Mol Sci.

[37] Heusch G., Andreadou I., Bell R. (2023). Health position paper and redox perspectives on reactive oxygen species as signals and targets of cardioprotection. Redox Biol.

[38] Vita J.A., Treasure C.B., Nabel E.G. (1990). Coronary vasomotor response to acetylcholine relates to risk factors for coronary artery disease. Circulation.

[39] Panza J.A., Quyyumi A.A., Brush J.E., Epstein S.E. (1990). Abnormal endothelium-dependent vascular relaxation in patients with essential hypertension. N Engl J Med.

[40] Gokce N., Keaney J.F., Hunter L.M., Watkins M.T., Menzoian J.O., Vita J.A. (2002). Risk stratification for postoperative cardiovascular events via noninvasive assessment of endothelial function: a prospective study. Circulation.

[41] Gokce N., Keaney J.F., Hunter L.M. (2003). Predictive value of noninvasively determined endothelial dysfunction for long-term cardiovascular events in patients with peripheral vascular disease. J Am Coll Cardiol.

[42] Calver A., Collier J., Vallance P. (1992). Inhibition and stimulation of nitric oxide synthesis in the human forearm arterial bed of patients with insulin-dependent diabetes. J Clin Invest.

[43] de Jager J., Dekker J.M., Kooy A. (2006). Endothelial dysfunction and low-grade inflammation explain much of the excess cardiovascular mortality in individuals with type 2 diabetes: the Hoorn Study. Arterioscler Thromb Vasc Biol.

[44] Heitzer T., Krohn K., Albers S., Meinertz T. (2000). Tetrahydrobiopterin improves endothelium-dependent vasodilation by increasing nitric oxide activity in patients with Type II diabetes mellitus. Diabetologia.

[45] Heitzer T., Finckh B., Albers S., Krohn K., Kohlschutter A., Meinertz T. (2001). Beneficial effects of alpha-lipoic acid and ascorbic acid on endothelium-dependent, nitric oxide-mediated vasodilation in diabetic patients: relation to parameters of oxidative stress. Free Radic Biol Med.

[46] Steven S., Oelze M., Hanf A. (2017). The SGLT2 inhibitor empagliflozin improves the primary diabetic complications in ZDF rats. Redox Biol.

[47] Giugliano D., Ceriello A., Paolisso G. (1996). Oxidative stress and diabetic vascular complications. Diabetes Care.

[48] Greene D.A., Lattimer S.A., Sima A.A. (1987). Sorbitol, phosphoinositides, and sodium-potassium-ATPase in the pathogenesis of diabetic complications. N Engl J Med.

[49] Goldin A., Beckman J.A., Schmidt A.M., Creager M.A. (2006). Advanced glycation end products: sparking the development of diabetic vascular injury. Circulation.

[50] Chawla D., Bansal S., Banerjee B.D., Madhu S.V., Kalra O.P., Tripathi A.K. (2014). Role of advanced glycation end product (AGE)-induced receptor (RAGE) expression in diabetic vascular complications. Microvasc Res.

[51] Nishikawa T., Edelstein D., Du X.L. (2000). Normalizing mitochondrial superoxide production blocks three pathways of hyperglycaemic damage. Nature.

[52] Uitto J., Perejda A.J., Grant G.A., Rowold E.A., Kilo C., Williamson J.R. (1982). Glycosylation of human glomerular basement membrane collagen: increased content of hexose in ketoamine linkage and unaltered hydroxylysine-O-glycosides in patients with diabetes. Connect Tissue Res.

[53] Bucala R., Tracey K.J., Cerami A. (1991). Advanced glycosylation products quench nitric oxide and mediate defective endothelium-dependent vasodilatation in experimental diabetes. J Clin Invest.

[54] Wautier M.P., Chappey O., Corda S., Stern D.M., Schmidt A.M., Wautier J.L. (2001). Activation of NADPH oxidase by AGE links oxidant stress to altered gene expression via RAGE. Am J Physiol Endocrinol Metab.

[55] Coughlan M.T., Thorburn D.R., Penfold S.A. (2009). RAGE-induced cytosolic ROS promote mitochondrial superoxide generation in diabetes. J Am Soc Nephrol.

[56] Bucciarelli L.G., Wendt T., Qu W. (2002). RAGE blockade stabilizes established atherosclerosis in diabetic apolipoprotein E-null mice. Circulation.

[57] Brownlee M. (2001). Biochemistry and molecular cell biology of diabetic complications. Nature.

[58] Yamagishi S., Nakamura K., Matsui T., Noda Y., Imaizumi T. (2008). Receptor for advanced glycation end products (RAGE): a novel therapeutic target for diabetic vascular complication. Curr Pharm Des.

[59] Sanajou D., Ghorbani Haghjo A., Argani H., Aslani S. (2018). AGE-RAGE axis blockade in diabetic nephropathy: Current status and future directions. Eur J Pharmacol.

[60] Wang X., Wang Q., Wei D., Chang X. (2021). Association between soluble receptor for advanced glycation end product and endogenous secretory soluble receptor for advanced glycation end product levels and carotid atherosclerosis in diabetes: a systematic review and meta-analysis. Can J Diabetes.

[61] Heitzer T., Schlinzig T., Krohn K., Meinertz T., Munzel T. (2001). Endothelial dysfunction, oxidative stress, and risk of cardiovascular events in patients with coronary artery disease. Circulation.

[62] Harrison D., Griendling K.K., Landmesser U., Hornig B., Drexler H. (2003). Role of oxidative stress in atherosclerosis. Am J Cardiol.

[63] van Drie R.W.A., van de Wouw J., Zandbergen L.M. (2024). Vasodilator reactive oxygen species ameliorate perturbed myocardial oxygen delivery in exercising swine with multiple comorbidities. Basic Res Cardiol.

[64] Schottker B., Brenner H., Jansen E.H. (2015). Evidence for the free radical/oxidative stress theory of ageing from the CHANCES consortium: a meta-analysis of individual participant data. BMC Med.

[65] Jay D., Hitomi H., Griendling K.K. (2006). Oxidative stress and diabetic cardiovascular complications. Free Radic Biol Med.

[66] Thomas M.C., Woodward M., Li Q. (2018). Relationship between plasma 8-OH-deoxyguanosine and cardiovascular disease and survival in type 2 diabetes mellitus: results from the ADVANCE trial. J Am Heart Assoc.

[67] Kjaer L.K., Cejvanovic V., Henriksen T. (2017). Cardiovascular and all-cause mortality risk associated with urinary excretion of 8-oxoGuo, a biomarker for RNA oxidation, in patients with type 2 diabetes: a prospective cohort study. Diabetes Care.

[68] Strohm L., Daiber A., Ubbens H. (2024). Role of inflammatory signaling pathways involving the CD40-CD40L-TRAF cascade in diabetes and hypertension-insights from animal and human studies. Basic Res Cardiol.

[69] Kaptoge S., Seshasai S.R., Gao P. (2014). Inflammatory cytokines and risk of coronary heart disease: new prospective study and updated meta-analysis. Eur Heart J.

[70] Ridker P.M., Rifai N., Rose L., Buring J.E., Cook N.R. (2002). Comparison of C-reactive protein and low-density lipoprotein cholesterol levels in the prediction of first cardiovascular events. N Engl J Med.

[71] Ridker P.M., MacFadyen J.G., Everett B.M. (2018). Relationship of C-reactive protein reduction to cardiovascular event reduction following treatment with canakinumab: a secondary analysis from the CANTOS randomised controlled trial. Lancet.

[72] Henry R.M., Ferreira I., Kostense P.J. (2004). Type 2 diabetes is associated with impaired endothelium-dependent, flow-mediated dilation, but impaired glucose metabolism is not; The Hoorn Study. Comparative Study. Atherosclerosis.

[73] Muzammil K., Khaleel A.Q., Merza M.S. (2024). The effects of omega-3 fatty acids on inflammatory and oxidative stress markers in patients with Type 2 diabetes mellitus: a systematic review and meta-analysis of controlled trials. Prostaglandins Other Lipid Mediat.

[74] Steven S., Munzel T., Daiber A. (2015). Exploiting the pleiotropic antioxidant effects of established drugs in cardiovascular disease. Int J Mol Sci.

[75] Harrison D.G., Guzik T.J., Lob H.E. (2011). Inflammation, immunity, and hypertension. Hypertension.

[76] Drexler H., Hornig B. (1999). Endothelial dysfunction in human disease. J Mol Cell Cardiol.

[77] Warnholtz A., Munzel T. (2000). Why do antioxidants fail to provide clinical benefit?. Curr Control Trials Cardiovasc Med.

[78] Porsti I., Bara A.T., Busse R., Hecker M. (1994). Release of nitric oxide by angiotensin-(1-7) from porcine coronary endothelium: implications for a novel angiotensin receptor. Br J Pharmacol.

[79] Mollnau H., Oelze M., August M. (2005). Mechanisms of increased vascular superoxide production in an experimental model of idiopathic dilated cardiomyopathy. Arterioscler Thromb Vasc Biol.

[80] Williams H.C., Griendling K.K. (2007). NADPH oxidase inhibitors: new antihypertensive agents?. J Cardiovasc Pharmacol.

[81] Caspritz G., Alpermann H.G., Schleyerbach R. (1986). Influence of the new angiotensin converting enzyme inhibitor ramipril on several models of acute inflammation and the adjuvant arthritis in the rat. Arzneimittel-Forschung.

[82] Takemoto M., Liao J.K. (2001). Pleiotropic effects of 3-hydroxy-3-methylglutaryl coenzyme a reductase inhibitors. Arterioscler Thromb Vasc Biol.

[83] Ray K.K., Cannon C.P. (2004). Pathological changes in acute coronary syndromes: the role of statin therapy in the modulation of inflammation, endothelial function and coagulation. J Thromb Thrombolysis.

[84] Patel T.N., Shishehbor M.H., Bhatt D.L. (2007). A review of high-dose statin therapy: targeting cholesterol and inflammation in atherosclerosis. Eur Heart J.

[85] Adam O., Laufs U. (2014). Rac1-mediated effects of HMG-CoA reductase inhibitors (statins) in cardiovascular disease. Antioxid Redox Signal.

[86] Wenzel P., Daiber A., Oelze M. (2008). Mechanisms underlying recoupling of eNOS by HMG-CoA reductase inhibition in a rat model of streptozotocin-induced diabetes mellitus. Atherosclerosis.

[87] Margaritis M., Channon K.M., Antoniades C. (2014). Statins as regulators of redox state in the vascular endothelium: beyond lipid lowering. Antioxid Redox Signal.

[88] Habeos I.G., Ziros P.G., Chartoumpekis D., Psyrogiannis A., Kyriazopoulou V., Papavassiliou A.G. (2008). Simvastatin activates Keap1/Nrf2 signaling in rat liver. J Mol Med.

[89] Ali F., Zakkar M., Karu K. (2009). Induction of the cytoprotective enzyme heme oxygenase-1 by statins is enhanced in vascular endothelium exposed to laminar shear stress and impaired by disturbed flow. J Biol Chem.

[90] Hibbert B., Simard T., Ramirez F.D. (2013). The effect of statins on circulating endothelial progenitor cells in humans: a systematic review. J Cardiovasc Pharmacol.

[91] Efentakis P., Choustoulaki A., Kwiatkowski G. (2025). Early microvascular coronary endothelial dysfunction precedes pembrolizumab-induced cardiotoxicity. Preventive role of high dose of atorvastatin. Basic Res Cardiol.

[92] Matsubara J., Sugiyama S., Sugamura K. (2012). A dipeptidyl peptidase-4 inhibitor, des-fluoro-sitagliptin, improves endothelial function and reduces atherosclerotic lesion formation in apolipoprotein E-deficient mice. J Am Coll Cardiol.

[93] Ussher J.R., Drucker D.J. (2012). Cardiovascular biology of the incretin system. Endocr Rev.

[94] Shah Z., Kampfrath T., Deiuliis J.A. (2011). Long-term dipeptidyl-peptidase 4 inhibition reduces atherosclerosis and inflammation via effects on monocyte recruitment and chemotaxis. Circulation.

[95] Burgmaier M., Liberman A., Mollmann J. (2013). Glucagon-like peptide-1 (GLP-1) and its split products GLP-1(9-37) and GLP-1(28-37) stabilize atherosclerotic lesions in apoe(-)/(-) mice. Atherosclerosis.

[96] Gaspari T., Liu H., Welungoda I. (2011). A GLP-1 receptor agonist liraglutide inhibits endothelial cell dysfunction and vascular adhesion molecule expression in an ApoE-/- mouse model. Diab Vasc Dis Res.

[97] Rakipovski G., Rolin B., Nohr J. (2018). The GLP-1 analogs liraglutide and semaglutide reduce atherosclerosis in ApoE(-/-) and LDLr(-/-) mice by a mechanism that includes inflammatory pathways. JACC Basic Transl Sci.

[98] Wenzel P., Knorr M., Kossmann S. (2011). Lysozyme M-positive monocytes mediate angiotensin II-induced arterial hypertension and vascular dysfunction. Circulation.

[99] Buldak L., Labuzek K., Buldak R.J., Machnik G., Boldys A., Okopien B. (2015). Exenatide (a GLP-1 agonist) improves the antioxidative potential of in vitro cultured human monocytes/macrophages. Naunyn Schmiedebergs Arch Pharmacol.

[100] Arakawa M., Mita T., Azuma K. (2010). Inhibition of monocyte adhesion to endothelial cells and attenuation of atherosclerotic lesion by a glucagon-like peptide-1 receptor agonist, exendin-4. Diabetes.

[101] Erdogdu O., Nathanson D., Sjoholm A., Nystrom T., Zhang Q. (2010). Exendin-4 stimulates proliferation of human coronary artery endothelial cells through eNOS-, PKA- and PI3K/Akt-dependent pathways and requires GLP-1 receptor. Mol Cell Endocrinol.

[102] Ban K., Noyan-Ashraf M.H., Hoefer J., Bolz S.S., Drucker D.J., Husain M. (2008). Cardioprotective and vasodilatory actions of glucagon-like peptide 1 receptor are mediated through both glucagon-like peptide 1 receptor-dependent and -independent pathways. Comparative Study. Circulation.

[103] Green B.D., Hand K.V., Dougan J.E., McDonnell B.M., Cassidy R.S., Grieve D.J. (2008). GLP-1 and related peptides cause concentration-dependent relaxation of rat aorta through a pathway involving KATP and cAMP. Arch Biochem Biophys.

[104] Shah Z., Pineda C., Kampfrath T. (2011). Acute DPP-4 inhibition modulates vascular tone through GLP-1 independent pathways. Vascul Pharmacol.

[105] Kroller-Schon S., Knorr M., Hausding M. (2012). Glucose-independent improvement of vascular dysfunction in experimental sepsis by dipeptidyl-peptidase 4 inhibition. Cardiovasc Res.

[106] Wang R., Lu L., Guo Y. (2015). Effect of Glucagon-like peptide-1 on high-glucose-induced oxidative stress and cell apoptosis in human endothelial cells and its underlying mechanism. J Cardiovasc Pharmacol.

[107] Jonik S., Marchel M., Grabowski M., Opolski G., Mazurek T. (2022). Gastrointestinal incretins-glucose-dependent insulinotropic polypeptide (GIP) and glucagon-like peptide-1 (GLP-1) beyond pleiotropic physiological effects are involved in pathophysiology of atherosclerosis and coronary artery disease-state of the art. Biology (Basel).

[108] Ussher J.R., Drucker D.J. (2023). Glucagon-like peptide 1 receptor agonists: cardiovascular benefits and mechanisms of action. Nat Rev Cardiol.

[109] Wang X., Yang X., Qi X. (2024). Anti-atherosclerotic effect of incretin receptor agonists. Front Endocrinol (Lausanne).

[110] Park B., Bakbak E., Teoh H. (2024). GLP-1 receptor agonists and atherosclerosis protection: the vascular endothelium takes center stage. Am J Physiol Heart Circ Physiol.

[111] Oeseburg H., de Boer R.A., Buikema H., van der Harst P., van Gilst W.H., Sillje H.H. (2010). Glucagon-like peptide 1 prevents reactive oxygen species-induced endothelial cell senescence through the activation of protein kinase A. Arterioscler Thromb Vasc Biol.

[112] Akarte A.S., Srinivasan B.P., Gandhi S., Sole S. (2012). Chronic DPP-IV inhibition with PKF-275-055 attenuates inflammation and improves gene expressions responsible for insulin secretion in streptozotocin induced diabetic rats. Eur J Pharm Sci.

[113] Batchuluun B., Inoguchi T., Sonoda N. (2014). Metformin and liraglutide ameliorate high glucose-induced oxidative stress via inhibition of PKC-NAD(P)H oxidase pathway in human aortic endothelial cells. Atherosclerosis.

[114] Shiraki A., Oyama J., Komoda H. (2012). The glucagon-like peptide 1 analog liraglutide reduces TNF-alpha-induced oxidative stress and inflammation in endothelial cells. Atherosclerosis.

[115] Chinda K., Palee S., Surinkaew S., Phornphutkul M., Chattipakorn S., Chattipakorn N. (2013). Cardioprotective effect of dipeptidyl peptidase-4 inhibitor during ischemia-reperfusion injury. Int J Cardiol.

[116] Inthachai T., Lekawanvijit S., Kumfu S. (2015). Dipeptidyl peptidase-4 inhibitor improves cardiac function by attenuating adverse cardiac remodelling in rats with chronic myocardial infarction. Exp Physiol.

[117] Bao W., Morimoto K., Hasegawa T. (2014). Orally administered dipeptidyl peptidase-4 inhibitor (alogliptin) prevents abdominal aortic aneurysm formation through an antioxidant effect in rats. J Vasc Surg.

[118] Abdelsalam R.M., Safar M.M. (2015). Neuroprotective effects of vildagliptin in rat rotenone Parkinson's disease model: role of RAGE-NFkappaB and Nrf2-antioxidant signaling pathways. J Neurochem.

[119] Salcedo I., Tweedie D., Li Y., Greig N.H. (2012). Neuroprotective and neurotrophic actions of glucagon-like peptide-1: an emerging opportunity to treat neurodegenerative and cerebrovascular disorders. Br J Pharmacol.

[120] Steven S., Hausding M., Kroller-Schon S. (2015). Gliptin and GLP-1 analog treatment improves survival and vascular inflammation/dysfunction in animals with lipopolysaccharide-induced endotoxemia. Basic Res Cardiol.

[121] Ramos H., Bogdanov P., Huerta J., Deas-Just A., Hernandez C., Simo R. (2022). Antioxidant effects of DPP-4 inhibitors in early stages of experimental diabetic retinopathy. Antioxidants.

[122] Shah P., Ardestani A., Dharmadhikari G. (2013). The DPP-4 inhibitor linagliptin restores beta-cell function and survival in human isolated islets through GLP-1 stabilization. J Clin Endocrinol Metab.

[123] Rizzo M.R., Barbieri M., Marfella R., Paolisso G. (2012). Reduction of oxidative stress and inflammation by blunting daily acute glucose fluctuations in patients with type 2 diabetes: role of dipeptidyl peptidase-IV inhibition. Diabetes Care.

[124] Martin M., Huguet J., Centelles J.J., Franco R. (Nov 15 1995). Expression of ecto-adenosine deaminase and CD26 in human T cells triggered by the TCR-CD3 complex. Possible role of adenosine deaminase as costimulatory molecule. J Immunol.

[125] Kameoka J., Tanaka T., Nojima Y., Schlossman S.F., Morimoto C. (1993). Direct association of adenosine deaminase with a T cell activation antigen, CD26. Science.

[126] Zhong J., Rao X., Rajagopalan S. (2013). An emerging role of dipeptidyl peptidase 4 (DPP4) beyond glucose control: potential implications in cardiovascular disease. Atherosclerosis.

[127] Amori R.E., Lau J., Pittas A.G. (2007). Efficacy and safety of incretin therapy in type 2 diabetes: systematic review and meta-analysis. JAMA.

[128] Baggio L.L., Varin E.M., Koehler J.A. (2020). Plasma levels of DPP4 activity and sDPP4 are dissociated from inflammation in mice and humans. Nat Commun.

[129] Helmstadter J., Frenis K., Filippou K. (2020). Endothelial GLP-1 (glucagon-like peptide-1) receptor mediates cardiovascular protection by liraglutide in mice with experimental arterial hypertension. Arterioscler Thromb Vasc Biol.

[130] Ravassa S., Beaumont J., Huerta A. (2015). Association of low GLP-1 with oxidative stress is related to cardiac disease and outcome in patients with type 2 diabetes mellitus: a pilot study. Free Radic Biol Med.

[131] Lambadiari V., Pavlidis G., Kousathana F. (2018). Effects of 6-month treatment with the glucagon like peptide-1 analogue liraglutide on arterial stiffness, left ventricular myocardial deformation and oxidative stress in subjects with newly diagnosed type 2 diabetes. Cardiovasc Diabetol.

[132] Fisslthaler B., Fleming I. (2009). Activation and signaling by the AMP-activated protein kinase in endothelial cells. Circ Res.

[133] Balteau M., Van Steenbergen A., Timmermans A.D. (2014). AMPK activation by glucagon-like peptide-1 prevents NADPH oxidase activation induced by hyperglycemia in adult cardiomyocytes. Am J Physiol Heart Circ Physiol.

[134] Helmstadter J., Keppeler K., Kuster L., Munzel T., Daiber A., Steven S. (2022). Glucagon-like peptide-1 (GLP-1) receptor agonists and their cardiovascular benefits-The role of the GLP-1 receptor. Br J Pharmacol.

[135] Armstrong M.J., Gaunt P., Aithal G.P. (2016). Liraglutide safety and efficacy in patients with non-alcoholic steatohepatitis (LEAN): a multicentre, double-blind, randomised, placebo-controlled phase 2 study. Lancet.

[136] Zheng R.H., Zhang W.W., Ji Y.N. (2020). Exogenous supplement of glucagon like peptide-1 protects the heart against aortic banding induced myocardial fibrosis and dysfunction through inhibiting mTOR/p70S6K signaling and promoting autophagy. Eur J Pharmacol.

[137] Tate M., Robinson E., Green B.D., McDermott B.J., Grieve D.J. (2016). Exendin-4 attenuates adverse cardiac remodelling in streptozocin-induced diabetes via specific actions on infiltrating macrophages. Basic Res Cardiol.

[138] Barale C., Buracco S., Cavalot F., Frascaroli C., Guerrasio A., Russo I. (2017). Glucagon-like peptide 1-related peptides increase nitric oxide effects to reduce platelet activation. Thromb Haemost.

[139] Sternkopf M., Nagy M., Baaten C. (2020). Native, intact glucagon-like peptide 1 is a natural suppressor of thrombus growth under physiological flow conditions. Arterioscler Thromb Vasc Biol.

[140] Cameron-Vendrig A., Reheman A., Siraj M.A. (2016). Glucagon-like peptide 1 receptor activation attenuates platelet aggregation and thrombosis. Diabetes.

[141] Steven S., Jurk K., Kopp M. (2017). Glucagon-like peptide-1 receptor signalling reduces microvascular thrombosis, nitro-oxidative stress and platelet activation in endotoxaemic mice. Br J Pharmacol.

[142] Jia G., Aroor A.R., Sowers J.R. (2016). Glucagon-like peptide 1 receptor activation and platelet function: beyond glycemic control. Diabetes.

[143] Loganathan J., Cohen A.C., Kaloupis G.M. (2022). A pilot clinical study to Evaluate Liraglutide-mediated Anti-platelet activity in patients with type-2 Diabetes (ELAID study). J Diabetes Complications.

[144] Zhang Y., Chen R., Jia Y., Chen M., Shuai Z. (2021). Effects of exenatide on coagulation and platelet aggregation in patients with type 2 diabetes. Drug Des Dev Ther.

[145] Pignatelli P., Baratta F., Buzzetti R. (2022). The sodium-glucose co-transporter-2 (SGLT2) inhibitors reduce platelet activation and thrombus formation by lowering NOX2-related oxidative stress: a pilot study. Antioxidants.

[146] Foer D., Amin T., Nagai J. (2023). Glucagon-like peptide-1 receptor pathway attenuates platelet activation in aspirin-exacerbated respiratory disease. J Immunol.

[147] Tsao C.W., Aday A.W., Almarzooq Z.I. (2023). Heart disease and stroke statistics-2023 update: a report from the American Heart Association. Circulation.

[148] Schafer A., Konig T., Bauersachs J., Akin M. (2022). Novel therapeutic strategies to reduce reperfusion injury after acute myocardial infarction. Curr Probl Cardiol.

[149] Heusch G., Gersh B.J. (2017). The pathophysiology of acute myocardial infarction and strategies of protection beyond reperfusion: a continual challenge. Eur Heart J.

[150] Heusch G. (2020). Myocardial ischaemia-reperfusion injury and cardioprotection in perspective. Nat Rev Cardiol.

[151] Timmers L., Henriques J.P., de Kleijn D.P. (2009). Exenatide reduces infarct size and improves cardiac function in a porcine model of ischemia and reperfusion injury. J Am Coll Cardiol.

[152] Ma X., Liu Z., Ilyas I. (2021). GLP-1 receptor agonists (GLP-1RAs): cardiovascular actions and therapeutic potential. Int J Biol Sci.

[153] Pandey S., Mangmool S., Parichatikanond W. (2023). Multifaceted roles of GLP-1 and Its analogs: a review on molecular mechanisms with a cardiotherapeutic perspective. Pharmaceuticals.

[154] Heusch G. (2024). Myocardial ischemia/reperfusion: Translational pathophysiology of ischemic heart disease. Med.

[155] Siraj M.A., Mundil D., Beca S. (2020). Cardioprotective GLP-1 metabolite prevents ischemic cardiac injury by inhibiting mitochondrial trifunctional protein-alpha. J Clin Invest.

[156] Sonne D.P., Engstrom T., Treiman M. (2008). Protective effects of GLP-1 analogues exendin-4 and GLP-1(9-36) amide against ischemia-reperfusion injury in rat heart. Regul Pept.

[157] Yellon D.M., Beikoghli Kalkhoran S., Davidson S.M. (2023). The RISK pathway leading to mitochondria and cardioprotection: how everything started. Basic Res Cardiol.

[158] Mangmool S., Hemplueksa P., Parichatikanond W., Chattipakorn N. (2015). Epac is required for GLP-1R-mediated inhibition of oxidative stress and apoptosis in cardiomyocytes. Mol Endocrinol.

[159] Nuamnaichati N., Mangmool S., Chattipakorn N., Parichatikanond W. (2020). Stimulation of GLP-1 receptor inhibits methylglyoxal-induced mitochondrial dysfunctions in H9c2 cardiomyoblasts: potential role of Epac/PI3K/Akt pathway. Front Pharmacol.

[160] Chen J., Xu S., Zhou W., Wu L., Wang L., Li W. (2020). Exendin-4 reduces ventricular arrhythmia activity and calcium sparks-mediated sarcoplasmic reticulum Ca leak in rats with heart failure. Int Heart J.

[161] Chen W.R., Tian F., Chen Y.D. (2016). Effects of liraglutide on no-reflow in patients with acute ST-segment elevation myocardial infarction. Int J Cardiol.

[162] Ravassa S., Zudaire A., Diez J. (2012). GLP-1 and cardioprotection: from bench to bedside. Cardiovasc Res.

[163] Ang R., Mastitskaya S., Hosford P.S. (2018). Modulation of cardiac ventricular excitability by GLP-1 (glucagon-like peptide-1). Circ Arrhythm Electrophysiol.

[164] Chang M.W., Chen C.H., Chen Y.C. (2015). Sitagliptin protects rat kidneys from acute ischemia-reperfusion injury via upregulation of GLP-1 and GLP-1 receptors. Acta Pharmacol Sin.

[165] Evans J.L., Goldfine I.D. (2013). Aging and insulin resistance: just say iNOS. Diabetes.

[166] Ferrannini E., Muscelli E., Frascerra S. (2014). Metabolic response to sodium-glucose cotransporter 2 inhibition in type 2 diabetic patients. J Clin Invest.

[167] Nan J., Wang D., Zhong R. (2024). Sodium glucose cotransporter2 inhibitors for type 1 diabetes mellitus: A meta-analysis of randomized controlled trials. Prim Care Diabetes.

[168] Oelze M., Kroller-Schon S., Welschof P. (2014). The sodium-glucose co-transporter 2 inhibitor empagliflozin improves diabetes-induced vascular dysfunction in the streptozotocin diabetes rat model by interfering with oxidative stress and glucotoxicity. PLoS One.

[169] Kumar S., Prasad S., Sitasawad S.L. (2013). Multiple antioxidants improve cardiac complications and inhibit cardiac cell death in streptozotocin-induced diabetic rats. PLoS One.

[170] Youssef M.E., Yahya G., Popoviciu M.S., Cavalu S., Abd-Eldayem M.A., Saber S. (2023). Unlocking the full potential of SGLT2 inhibitors: expanding applications beyond glycemic control. Int J Mol Sci.

[171] Oe Y., Vallon V. (2022). The pathophysiological basis of diabetic kidney protection by inhibition of SGLT2 and SGLT1. Kidney Dial.

[172] Bierhaus A., Fleming T., Stoyanov S. (2012). Methylglyoxal modification of Nav1.8 facilitates nociceptive neuron firing and causes hyperalgesia in diabetic neuropathy. Nat Med.

[173] Wautier J.L., Wautier M.P., Schmidt A.M. (1994). Advanced glycation end products (AGEs) on the surface of diabetic erythrocytes bind to the vessel wall via a specific receptor inducing oxidant stress in the vasculature: a link between surface-associated AGEs and diabetic complications. Proc Natl Acad Sci USA.

[174] Larsen E.L., Andersen A., Kjaer L.K. (2023). Effects of two- and twelve-weeks sodium-glucose cotransporter 2 inhibition on DNA and RNA oxidation: two randomized, placebo-controlled trials. Free Radic Res.

[175] Prochaska J.H., Junger C., Schulz A. (2023). Effects of empagliflozin on left ventricular diastolic function in addition to usual care in individuals with type 2 diabetes mellitus-results from the randomized, double-blind, placebo-controlled EmDia trial. Clin Res Cardiol.

[176] Vincent R.K., Williams D.M., Evans M. (2020). A look to the future in non-alcoholic fatty liver disease: Are glucagon-like peptide-1 analogues or sodium-glucose co-transporter-2 inhibitors the answer?. Diabetes Obes Metab.

[177] Shinozaki S., Tahara T., Lefor A.K., Ogura M. (2020). Long-term empagliflozin therapy improves levels of hepatic fibrosis marker in patients with non-alcoholic fatty liver disease complicated by type 2 diabetes mellitus. J Med Invest.

[178] Taheri H., Malek M., Ismail-Beigi F. (2020). Effect of empagliflozin on liver steatosis and fibrosis in patients with non-alcoholic fatty liver disease without diabetes: a randomized, double-blind, placebo-controlled trial. Adv Ther.

[179] Jojima T., Tomotsune T., Iijima T., Akimoto K., Suzuki K., Aso Y. (2016). Empagliflozin (an SGLT2 inhibitor), alone or in combination with linagliptin (a DPP-4 inhibitor), prevents steatohepatitis in a novel mouse model of non-alcoholic steatohepatitis and diabetes. Diabetol Metab Syndr.

[180] Zelniker T.A., Braunwald E. (2020). Mechanisms of cardiorenal effects of sodium-glucose cotransporter 2 inhibitors: JACC state-of-the-art review. J Am Coll Cardiol.

[181] Nikolaou P.E., Mylonas N., Makridakis M. (2022). Cardioprotection by selective SGLT-2 inhibitors in a non-diabetic mouse model of myocardial ischemia/reperfusion injury: a class or a drug effect?. Basic Res Cardiol.

[182] Nikolaou P.E., Efentakis P., Abu Qourah F. (2021). Chronic empagliflozin treatment reduces myocardial infarct size in nondiabetic mice through STAT-3-mediated protection on microvascular endothelial cells and reduction of oxidative stress. Antioxid Redox Signal.

[183] Andreadou I., Bell R.M., Botker H.E., Zuurbier C.J. (2020). SGLT2 inhibitors reduce infarct size in reperfused ischemic heart and improve cardiac function during ischemic episodes in preclinical models. Biochim Biophys Acta Mol Basis Dis.

[184] Basalay M.V., Korsak A., He Z., Gourine A.V., Davidson S.M., Yellon D.M. (2025). SGLT2 inhibition induces cardioprotection by increasing parasympathetic activity. Circ Res.

[185] Chen S., Wang Q., Christodoulou A. (2023). Sodium glucose cotransporter-2 inhibitor empagliflozin reduces infarct size independently of sodium glucose cotransporter-2. Circulation.

[186] Mylonas N., Nikolaou P.E., Karakasis P., Stachteas P., Fragakis N., Andreadou I. (2024). Endothelial protection by sodium-glucose cotransporter 2 inhibitors: a literature review of in vitro and in vivo studies. Int J Mol Sci.

[187] Patel D.K., Strong J. (2019). The pleiotropic effects of sodium-glucose cotransporter-2 inhibitors: beyond the glycemic benefit. Diabetes Ther.

[188] Tanna M.S., Goldberg L.R. (2021). The pleiotropic cardiovascular effects of sodium-glucose cotransporter-2 inhibitors. Curr Opin Cardiol.

[189] Rastogi A., Januzzi J.L. (2023). Pleiotropic effects of sodium-glucose cotransporter-2 inhibitors in cardiovascular disease and chronic kidney disease. J Clin Med.

[190] Theofilis P., Sagris M., Oikonomou E. (2022). Pleiotropic effects of SGLT2 inhibitors and heart failure outcomes. Diabetes Res Clin Pract.

[191] Cianciolo G., De Pascalis A., Gasperoni L. (2020). The off-target effects, electrolyte and mineral disorders of SGLT2i. Molecules.

[192] Nguyen T., Wen S., Gong M. (2020). Dapagliflozin activates neurons in the central nervous system and regulates cardiovascular activity by inhibiting SGLT-2 in mice. Diabetes Metab Syndr Obes.

[193] Forrester E.A., Benitez-Angeles M., Redford K.E. (2024). Crucial role for sensory nerves and Na/H exchanger inhibition in dapagliflozin- and empagliflozin-induced arterial relaxation. Cardiovasc Res.

[194] Mroueh A., Algara-Suarez P., Fakih W. (2025). SGLT2 expression in human vasculature and heart correlates with low-grade inflammation and causes eNOS-NO/ROS imbalance. Cardiovasc Res.

[195] Chang G.J., Chen W.J., Hsu Y.J., Chen Y.H. (2024). Empagliflozin attenuates neointima formation after arterial injury and inhibits smooth muscle cell proliferation and migration by suppressing platelet-derived growth factor-related signaling. J Am Heart Assoc.

[196] Behnammanesh G., Durante G.L., Khanna Y.P., Peyton K.J., Durante W. (2020). Canagliflozin inhibits vascular smooth muscle cell proliferation and migration: Role of heme oxygenase-1. Redox Biol.

[197] Sukhanov S., Higashi Y., Yoshida T. (2021). The SGLT2 inhibitor Empagliflozin attenuates interleukin-17A-induced human aortic smooth muscle cell proliferation and migration by targeting TRAF3IP2/ROS/NLRP3/Caspase-1-dependent IL-1beta and IL-18 secretion. Cell Signal.

[198] Uthman L., Nederlof R., Eerbeek O. (2019). Delayed ischaemic contracture onset by empagliflozin associates with NHE1 inhibition and is dependent on insulin in isolated mouse hearts. Cardiovasc Res.

[199] Santos-Gallego C.G., Requena-Ibanez J.A., San Antonio R. (2021). Empagliflozin ameliorates diastolic dysfunction and left ventricular fibrosis/stiffness in nondiabetic heart failure: a multimodality study. JACC Cardiovasc Imaging.

[200] Dasari D., Goyal S.G., Penmetsa A., Sriram D., Dhar A. (2023). Canagliflozin protects diabetic cardiomyopathy by mitigating fibrosis and preserving the myocardial integrity with improved mitochondrial function. Eur J Pharmacol.

[201] Ye Y., Bajaj M., Yang H.C., Perez-Polo J.R., Birnbaum Y. (2017). SGLT-2 Inhibition with dapagliflozin reduces the activation of the Nlrp3/ASC inflammasome and attenuates the development of diabetic cardiomyopathy in mice with type 2 diabetes. Further augmentation of the effects with saxagliptin, a DPP4 inhibitor. Cardiovasc Drugs Ther.

[202] Zhang Y., Lin X., Chu Y. (2021). Dapagliflozin: a sodium-glucose cotransporter 2 inhibitor, attenuates angiotensin II-induced cardiac fibrotic remodeling by regulating TGFbeta1/Smad signaling. Cardiovas Diabetol.

[203] Scisciola L., Taktaz F., Fontanella R.A. (2023). Targeting high glucose-induced epigenetic modifications at cardiac level: the role of SGLT2 and SGLT2 inhibitors. Cardiovasc Diabetol.

[204] Yokoi A., Asahara S.I., Inoue H. (2024). Dapagliflozin administration to a mouse model of type 2 diabetes induces DNA methylation and gene expression changes in pancreatic islets. Biochem Biophys Res Commun.

[205] Attia S.M., Albekairi N.A., Alshamrani A.A. (2024). Dapagliflozin suppresses diabetes-induced oxidative DNA damage and hypermethylation in mouse somatic cells. Mutat Res Genet Toxicol Environ Mutagen.

[206] Huang Q., Liu L., Tan X. (2024). Empagliflozin alleviates neuroinflammation by inhibiting astrocyte activation in the brain and regulating gut microbiota of high-fat diet mice. J Affect Disord.

[207] Wang L., Wang Y., Xu H., Li W. (2024). Effect of dapagliflozin on ferroptosis through the gut microbiota metabolite TMAO during myocardial ischemia-reperfusion injury in diabetes mellitus rats. Sci Rep.

[208] Liao L., Zhang L., Yang C. (2024). Sotagliflozin attenuates cardiac dysfunction and depression-like behaviors in mice with myocardial infarction through the gut-heart-brain axis. Neurobiol Dis.

[209] Lei L., Zhu T., Cui T.J. (2024). Renoprotective effects of empagliflozin in high-fat diet-induced obesity-related glomerulopathy by regulation of gut-kidney axis. Am J Physiol Cell Physiol.

[210] Yuan F., Zhang T., Jia S., Zhao J., Wan B., Liu G. (2024). Fine mapping-based multi-omics analysis interprets the gut-lung axis function of SGLT2 inhibitors. Front Cell Infect Microbiol.

[211] Heusch G., Kleinbongard P. (2025). The enigmata of cardioprotection with SGLT2 inhibition. JACC Basic Transl Sci.

[212] Nikolaou P.E., Konijnenberg L.S.F., Kostopoulos I.V. (Jan 2025). Empagliflozin in acute myocardial infarction reduces no-reflow and preserves cardiac function by preventing endothelial damage. JACC Basic Transl Sci.

[213] Heusch G. (Jan 13 2022). Coronary blood flow in heart failure: cause, consequence and bystander. Basic Res Cardiol.

[214] Braunwald E. (2022). Gliflozins in the management of cardiovascular disease. N Engl J Med.

[215] Kalyani R.R. (2021). Glucose-lowering drugs to reduce cardiovascular risk in type 2 diabetes. N Engl J Med.

[216] Marso S.P., Bain S.C., Consoli A. (2016). Semaglutide and cardiovascular outcomes in patients with type 2 diabetes. N Engl J Med.

[217] Marso S.P., Daniels G.H., Brown-Frandsen K. (2016). Liraglutide and cardiovascular outcomes in type 2 diabetes. N Engl J Med.

[218] Wiviott S.D., Raz I., Bonaca M.P. (2019). Dapagliflozin and cardiovascular outcomes in type 2 diabetes. N Engl J Med.

[219] Zinman B., Wanner C., Lachin J.M. (2015). Empagliflozin, cardiovascular outcomes, and mortality in type 2 diabetes. N Engl J Med.

[220] Abbott C.A., Yu D.M., Woollatt E., Sutherland G.R., McCaughan G.W., Gorrell M.D. (2000). Cloning, expression and chromosomal localization of a novel human dipeptidyl peptidase (DPP) IV homolog, DPP8. Eur J Biochem.

[221] Buhling F., Junker U., Reinhold D., Neubert K., Jager L., Ansorge S. (1995). Functional role of CD26 on human B lymphocytes. Immunol Lett.

[222] Buhling F., Kunz D., Reinhold D. (1994). Expression and functional role of dipeptidyl peptidase IV (CD26) on human natural killer cells. Nat Immun.

[223] Gorrell M.D. (2005). Dipeptidyl peptidase IV and related enzymes in cell biology and liver disorders. Clin Sci (Lond).

[224] Palmieri F.E., Ward P.E. (1989). Dipeptidyl(amino)peptidase IV and post proline cleaving enzyme in cultured endothelial and smooth muscle cells. Adv Exp Med Biol.

[225] Baggio L.L., Drucker D.J. (2007). Biology of incretins: GLP-1 and GIP. Review. Gastroenterology.

[226] Lund P.K., Goodman R.H., Dee P.C., Habener J.F. (1982). Pancreatic preproglucagon cDNA contains two glucagon-related coding sequences arranged in tandem. Proc Natl Acad Sci USA.

[227] Doyle M.E., Egan J.M. (Mar 2007). Mechanisms of action of glucagon-like peptide 1 in the pancreas. Pharmacol Therap.

[228] Nauck M.A. (2011). Incretin-based therapies for type 2 diabetes mellitus: properties, functions, and clinical implications. Am J Medicine.

[229] Brubaker P.L., Drucker D.J. (2002). Structure-function of the glucagon receptor family of G protein-coupled receptors: the glucagon, GIP, GLP-1, and GLP-2 receptors. A. Rec channel.

[230] Mentlein R., Gallwitz B., Schmidt W.E. (1993). Dipeptidyl-peptidase IV hydrolyses gastric inhibitory polypeptide, glucagon-like peptide-1(7-36)amide, peptide histidine methionine and is responsible for their degradation in human serum. Eur J Biochem.

[231] Kieffer T.J., McIntosh C.H., Pederson R.A. (1995). Degradation of glucose-dependent insulinotropic polypeptide and truncated glucagon-like peptide 1 in vitro and in vivo by dipeptidyl peptidase IV.

[232] Kern M., Kloting N., Niessen H.G. (2012). Linagliptin improves insulin sensitivity and hepatic steatosis in diet-induced obesity. PLoS One.

[233] Darsalia V., Ortsater H., Olverling A. (2013). The DPP-4 inhibitor linagliptin counteracts stroke in the normal and diabetic mouse brain: a comparison with glimepiride. Diabetes.

[234] Nishioka T., Shinohara M., Tanimoto N., Kumagai C., Hashimoto K. (2012). Sitagliptin, a dipeptidyl peptidase-IV inhibitor, improves psoriasis. Dermatology.

[235] Huang C.Y., Shih C.M., Tsao N.W. (2012). Dipeptidyl peptidase-4 inhibitor improves neovascularization by increasing circulating endothelial progenitor cells. Br J Pharmacol.

[236] Brenner C., Krankel N., Kuhlenthal S. (2014). Short-term inhibition of DPP-4 enhances endothelial regeneration after acute arterial injury via enhanced recruitment of circulating progenitor cells. Int J Cardiol.

[237] Fadini G.P., Boscaro E., Albiero M. (2010). The oral dipeptidyl peptidase-4 inhibitor sitagliptin increases circulating endothelial progenitor cells in patients with type 2 diabetes: possible role of stromal-derived factor-1alpha. Diabetes Care.

[238] Brenner C., Adrion C., Grabmaier U. (2016). Sitagliptin plus granulocyte colony-stimulating factor in patients suffering from acute myocardial infarction: a double-blind, randomized placebo-controlled trial of efficacy and safety (SITAGRAMI trial). Int J Cardiol.

[239] Xiao-Yun X., Zhao-Hui M., Ke C., Hong-Hui H., Yan-Hong X. (2011). Glucagon-like peptide-1 improves proliferation and differentiation of endothelial progenitor cells via upregulating VEGF generation. Med Sci Monit.

[240] Drucker D.J. (2024). Discovery of GLP-1-based drugs for the treatment of obesity. N Engl J Med.

[241] Drucker D.J. (2024). The benefits of GLP-1 drugs beyond obesity. Science.

[242] Drucker D.J. (2024). The GLP-1 journey: from discovery science to therapeutic impact. J Clin Invest.

[243] Drucker D.J. (2025). Author correction: GLP-1-based therapies for diabetes, obesity and beyond. Nat Rev Drug Discov.

[244] Nowak A., Bojanowska E. (2008). Effects of peripheral or central GLP-1 receptor blockade on leptin-induced suppression of appetite. J Physiol Pharmacol.

[245] Holmes G.M., Browning K.N., Tong M., Qualls-Creekmore E., Travagli R.A. (2009). Vagally mediated effects of glucagon-like peptide 1: in vitro and in vivo gastric actions. J Physiol.

[246] Saran A., Raisinghani R., Paliwal S., Sharma S. (2025). GLP-1R agonists: recent advances, current gaps, and future challenges. Mol Divers.

[247] Boyle J.G., Livingstone R., Petrie J.R. (2018). Cardiovascular benefits of GLP-1 agonists in type 2 diabetes: a comparative review. Clin Sci (Lond).

[248] Yaribeygi H., Maleki M., Jamialahmadi T., Sahebkar A. (2024). Anti-inflammatory benefits of semaglutide: state of the art. J Clin Transl Endocrinol.

[249] Perry R.J., Shulman G.I. (2020). Sodium-glucose cotransporter-2 inhibitors: understanding the mechanisms for therapeutic promise and persisting risks. J Biol Chem.

[250] Sawicki K.T., Ben-Sahra I., McNally E.M. (2021). SGLT2 inhibition on cardiac mitochondrial function: searching for a sweet spot. J Am Heart Assoc.

[251] Rieg T., Masuda T., Gerasimova M. (2014). Increase in SGLT1-mediated transport explains renal glucose reabsorption during genetic and pharmacological SGLT2 inhibition in euglycemia. Am J Physiol Renal Physiol.

[252] Yamamoto Y., Yamagishi S., Yonekura H. (2000). Roles of the AGE-RAGE system in vascular injury in diabetes. Ann N Y Acad Sci.

[253] Wanner C., Inzucchi S.E., Lachin J.M. (2016). Empagliflozin and progression of kidney disease in type 2 diabetes. N Engl J Med.

[254] Solomon S.D., McMurray J.J.V., Claggett B. (2022). Dapagliflozin in Heart Failure with Mildly Reduced or Preserved Ejection Fraction. N Engl J Med.

[255] Heerspink H.J.L., Stefansson B.V., Correa-Rotter R. (2020). Dapagliflozin in patients with chronic kidney disease. N Engl J Med.

[256] Cai X., Yang W., Gao X. (2018). The association between the dosage of SGLT2 inhibitor and weight reduction in type 2 diabetes patients: a meta-analysis. Obesity.

[257] Hu M., Cai X., Yang W., Zhang S., Nie L., Ji L. (2020). Effect of hemoglobin A1c reduction or weight reduction on blood pressure in glucagon-like peptide-1 receptor agonist and sodium-glucose cotransporter-2 inhibitor treatment in type 2 diabetes mellitus: a meta-analysis. J Am Heart Assoc.

[258] Cho Y.K., Kim Y.J., Jung C.H. (2021). Effect of Sodium-glucose cotransporter 2 inhibitors on weight reduction in overweight and obese populations without diabetes: a systematic review and a meta-analysis. J Obes Metab Syndr.

[259] Pfeffer M.A., Claggett B., Diaz R. (2015). Lixisenatide in patients with type 2 diabetes and acute coronary syndrome. N Engl J Med.

[260] Batlle J.V.I., Sweeck L., Wannijn J., Vandenhove H. (2016). Environmental risks of radioactive discharges from a low-level radioactive waste disposal site at Dessel, Belgium. J Environ Radioact.

[261] Gerstein H.C., Colhoun H.M., Dagenais G.R. (2019). Dulaglutide and cardiovascular outcomes in type 2 diabetes (REWIND): a double-blind, randomised placebo-controlled trial. Lancet.

[262] Gerstein H.C., Sattar N., Rosenstock J. (2021). Cardiovascular and renal outcomes with efpeglenatide in type 2 diabetes. N Engl J Med.

[263] Dost A., Hofer S., Herbst A. (2009). Factors contributing to terminal digital preference in 91,398 patients with diabetes mellitus in Germany and Austria: possible impact on therapeutic decisions. Diabetic Med.

[264] Gaspari T., Welungoda I., Widdop R.E., Simpson R.W., Dear A.E. (2013). The GLP-1 receptor agonist liraglutide inhibits progression of vascular disease via effects on atherogenesis, plaque stability and endothelial function in an ApoE(-/-) mouse model. Diabetes Vasc Dis Res.

[265] Copur S., Demiray A., Cherney D., Tuttle K., Kanbay M. (2023). Tirzepatide decreases systolic and diastolic blood pressure. Eur J Intern Med.

[266] Wu W., Tong H.M., Li Y.S., Cui J. (2024). The effect of semaglutide on blood pressure in patients with type-2 diabetes: a systematic review and meta-analysis. Endocrine.

[267] Perkovic V., Tuttle K.R., Rossing P. (2024). Effects of semaglutide on chronic kidney disease in patients with type 2 diabetes. N Engl J Med.

[268] Inzucchi S.E., Tan X., Liang Y., Yedigarova L., Xie L., de Havenon A. (2025). Cardiovascular events in adults with type 2 diabetes and ASCVD initiating once-weekly semaglutide vs DPP-4is in the USA. Diabetes Ther.

[269] Gerstein H.C., Hart R., Colhoun H.M. (2020). The effect of dulaglutide on stroke: an exploratory analysis of the REWIND trial. Lancet Diabetes Endocrinol.

[270] Nizari S., Basalay M., Chapman P. (2021). Glucagon-like peptide-1 (GLP-1) receptor activation dilates cerebral arterioles, increases cerebral blood flow, and mediates remote (pre)conditioning neuroprotection against ischaemic stroke. Basic Res Cardiol.

[271] Giugliano D., Longo M., Signoriello S. (2022). The effect of DPP-4 inhibitors, GLP-1 receptor agonists and SGLT-2 inhibitors on cardiorenal outcomes: a network meta-analysis of 23 CVOTs. Cardiovasc Diabetol.

[272] Neves J.S., Vasques-Novoa F., Borges-Canha M. (2023). Risk of adverse events with liraglutide in heart failure with reduced ejection fraction: a post hoc analysis of the FIGHT trial. Diabetes Obes Metab.

[273] Sharma A., Ambrosy A.P., DeVore A.D. (2018). Liraglutide and weight loss among patients with advanced heart failure and a reduced ejection fraction: insights from the FIGHT trial. ESC Heart Fail.

[274] Jorsal A., Kistorp C., Holmager P. (2017). Effect of liraglutide, a glucagon-like peptide-1 analogue, on left ventricular function in stable chronic heart failure patients with and without diabetes (LIVE)-a multicentre, double-blind, randomised, placebo-controlled trial. Eur J Heart Fail.

[275] Neves J.S., Packer M., Ferreira J.P. (2023). Increased risk of heart failure hospitalization with GLP-1 receptor agonists in patients with reduced ejection fraction: a meta-analysis of the EXSCEL and FIGHT trials. J Card Fail.

[276] Holman R.R., Bethel M.A., Mentz R.J. (2017). Effects of once-weekly exenatide on cardiovascular outcomes in type 2 diabetes. N Engl J Med.

[277] Kosiborod M.N., Petrie M.C., Borlaug B.A. (2024). Semaglutide in patients with obesity-related heart failure and type 2 diabetes. N Engl J Med.

[278] Kosiborod M.N., Abildstrom S.Z., Borlaug B.A. (2023). Semaglutide in patients with heart failure with preserved ejection fraction and obesity. N Engl J Med.

[279] Lincoff A.M., Brown-Frandsen K., Colhoun H.M. (2023). Semaglutide and cardiovascular outcomes in obesity without diabetes. N Engl J Med.

[280] Kosiborod M.N., Deanfield J., Pratley R. (2024). Semaglutide versus placebo in patients with heart failure and mildly reduced or preserved ejection fraction: a pooled analysis of the SELECT, FLOW, STEP-HFpEF, and STEP-HFpEF DM randomised trials. Lancet.

[281] Drucker D.J. (2024). Efficacy and safety of GLP-1 medicines for type 2 diabetes and obesity. Diabetes Care.

[282] Nauck M.A., Meier J.J., Cavender M.A., Abd El Aziz M., Drucker D.J. (2017). Cardiovascular actions and clinical outcomes with glucagon-like peptide-1 receptor agonists and dipeptidyl peptidase-4 inhibitors. Circulation.

[283] Sattar N., Lee M.M.Y., Kristensen S.L. (2021). Cardiovascular, mortality, and kidney outcomes with GLP-1 receptor agonists in patients with type 2 diabetes: a systematic review and meta-analysis of randomised trials. Lancet Diabetes Endocrinol.

[284] Hernandez A.F., Green J.B., Janmohamed S. (2018). Albiglutide and cardiovascular outcomes in patients with type 2 diabetes and cardiovascular disease (Harmony Outcomes): a double-blind, randomised placebo-controlled trial. Lancet.

[285] Scirica B.M., Bhatt D.L., Braunwald E. (2013). Saxagliptin and cardiovascular outcomes in patients with type 2 diabetes mellitus. N Engl J Med.

[286] Rosenstock J., Kahn S.E., Johansen O.E. (2019). Effect of linagliptin vs glimepiride on major adverse cardiovascular outcomes in patients with type 2 diabetes: the CAROLINA randomized clinical trial. JAMA.

[287] Zeng Y., Li C., Guan M. (2014). The DPP-4 inhibitor sitagliptin attenuates the progress of atherosclerosis in apolipoprotein-E-knockout mice via AMPK- and MAPK-dependent mechanisms. Cardiovasc Diabetol.

[288] Seferovic P.M., Petrie M.C., Filippatos G.S. (2018). Type 2 diabetes mellitus and heart failure: a position statement from the Heart Failure Association of the European Society of Cardiology. Eur J Heart Fail.

[289] Fadini G.P., Avogaro A., Degli Esposti L. (2015). Risk of hospitalization for heart failure in patients with type 2 diabetes newly treated with DPP-4 inhibitors or other oral glucose-lowering medications: a retrospective registry study on 127,555 patients from the Nationwide OsMed Health-DB Database. Eur Heart J.

[290] Filion K.B., Azoulay L., Platt R.W. (2016). A multicenter observational study of incretin-based drugs and heart failure. N Engl J Med.

[291] Marx N., Federici M., Schütt K. (2023). 2023 ESC Guidelines for the management of cardiovascular disease in patients with diabetes: Developed by the task force on the management of cardiovascular disease in patients with diabetes of the European Society of Cardiology (ESC). Eur Heart J.

[292] Patel S.M., Kang Y.M., Im K. (2024). Sodium-glucose cotransporter-2 inhibitors and major adverse cardiovascular outcomes: a SMART-C collaborative meta-analysis. Circulation.

[293] Neal B., Perkovic V., Mahaffey K.W. (2017). Canagliflozin and cardiovascular and renal events in type 2 diabetes. N Engl J Med.

[294] Perkovic V., Jardine M.J., Neal B. (2019). Canagliflozin and renal outcomes in type 2 diabetes and nephropathy. N Engl J Med.

[295] Cannon C.P., Pratley R., Dagogo-Jack S. (2020). Cardiovascular outcomes with ertugliflozin in type 2 diabetes. N Engl J Med.

[296] Packer M., Anker S.D., Butler J. (2020). Cardiovascular and renal outcomes with empagliflozin in heart failure. N Engl J Med.

[297] Anker S.D., Butler J., Filippatos G. (2021). Empagliflozin in heart failure with a preserved ejection fraction. N Engl J Med.

[298] McMurray J.J.V., Solomon S.D., Inzucchi S.E. (2019). Dapagliflozin in patients with heart failure and reduced ejection fraction. N Engl J Med.

[299] Empa-Kidney Collaborative Group, Herrington W.G., Staplin N. (2023). Empagliflozin in patients with chronic kidney disease. N Engl J Med.

[300] Bhatt D.L., Szarek M., Steg P.G. (Jan 14 2021). Sotagliflozin in patients with diabetes and recent worsening heart failure. N Engl J Med.

[301] Cosentino F., Cannon C.P., Cherney D.Z.I. (2020). Efficacy of ertugliflozin on heart failure-related events in patients with type 2 diabetes mellitus and established atherosclerotic cardiovascular disease: results of the VERTIS CV trial. Circulation.

[302] Berger J.H., Matsuura T.R., Bowman C.E. (2024). SGLT2 Inhibitors act independently of SGLT2 to confer benefit for HFrEF in mice. Circ Res.

[303] Neves J.S., Borges-Canha M., Vasques-Novoa F. (2023). GLP-1 receptor agonist therapy with and without SGLT2 inhibitors in patients with type 2 diabetes. J Am Coll Cardiol.

[304] Gerstein H.C., Branch K., Heenan L. (2021). Design and baseline characteristics of the AMPLITUDE-O cardiovascular outcomes trial of efpeglenatide, a weekly glucagon-like peptide-1 receptor agonist. DiabetesObes Metab.

[305] Frias J.P., Guja C., Hardy E. (2016). Exenatide once weekly plus dapagliflozin once daily versus exenatide or dapagliflozin alone in patients with type 2 diabetes inadequately controlled with metformin monotherapy (DURATION-8): a 28 week, multicentre, double-blind, phase 3, randomised controlled trial. Lancet Diabetes Endocrinol.

[306] Simms-Williams N., Treves N., Yin H. (2024). Effect of combination treatment with glucagon-like peptide-1 receptor agonists and sodium-glucose cotransporter-2 inhibitors on incidence of cardiovascular and serious renal events: population based cohort study. Bmj.

[307] Lambadiari V., Thymis J., Kouretas D. (2021). Effects of a 12-month treatment with glucagon-like peptide-1 receptor agonists, sodium-glucose cotransporter-2 inhibitors, and their combination on oxidant and antioxidant biomarkers in patients with type 2 diabetes. Antioxidants.

[308] Ikonomidis I., Pavlidis G., Thymis J. (2020). Effects of glucagon-like peptide-1 receptor agonists, sodium-glucose cotransporter-2 inhibitors, and their combination on endothelial glycocalyx, arterial function, and myocardial work index in patients with type 2 diabetes mellitus after 12-month treatment. J Am Heart Assoc.

[309] McGuire D.K., Marx N., Mulvagh S.L. (2025). Oral semaglutide and cardiovascular outcomes in high-risk type 2 diabetes. N Engl J Med.

[310] Ward Z.J., Bleich S.N., Long M.W., Gortmaker S.L. (2021). Association of body mass index with health care expenditures in the United States by age and sex. PLoS One.

[311] Apovian C.M. (2016). Obesity: definition, comorbidities, causes, and burden. Am J Manag Care.

[312] Guo X., Zhou Z., Lyu X. (2022). The antiobesity effect and safety of GLP-1 receptor agonist in overweight/obese patients without diabetes: a systematic review and meta-analysis. Horm Metab Res.

[313] Liao C., Liang X., Zhang X., Li Y. (2023). The effects of GLP-1 receptor agonists on visceral fat and liver ectopic fat in an adult population with or without diabetes and nonalcoholic fatty liver disease: a systematic review and meta-analysis. PLoS One.

[314] Idrees Z., Cancarevic I., Huang L. (2022). FDA-approved pharmacotherapy for weight loss over the last decade. Cureus.

[315] Wilding J.P.H., Batterham R.L., Calanna S. (2021). Once-weekly semaglutide in adults with overweight or obesity. N Engl J Med.

[316] Mehta A., Marso S.P., Neeland I.J. (2017). Liraglutide for weight management: a critical review of the evidence. Obes Sci Pract.

[317] McDonagh T.A., Metra M., Adamo M. (2021). 2021 ESC Guidelines for the diagnosis and treatment of acute and chronic heart failure. Eur Heart J.

[318] McDonagh T.A., Metra M., Adamo M. (2023). 2023 Focused update of the 2021 ESC guidelines for the diagnosis and treatment of acute and chronic heart failure. Eur Heart J.

[319] Davies M.J., Aroda V.R., Collins B.S. (2022). Management of hyperglycemia in type 2 diabetes, 2022. A consensus report by the American Diabetes Association (ADA) and the European Association for the Study of Diabetes (EASD). Diabetes Care.

[320] McCullough P.A., Kluger A.Y., Tecson K.M. (2018). Inhibition of the sodium-proton antiporter (exchanger) is a plausible mechanism of potential benefit and harm for drugs designed to block sodium glucose co-transporter 2. Rev Cardiovasc Med.

[321] Zhao X. Huang K. Zheng M. Duan J. Effect of liraglutide on blood pressure: a meta-analysis of liraglutide randomized controlled trials. BMC Endocr Disord 2019;19(1):4. 10.1186/s12902-018-0332-5 PMC6323665 30616638

[322] Wajdlich M., Nowicki M. (2024). The impact of GLP-1 receptor agonist liraglutide on blood pressure profile, hydration, natriuresis in diabetic patients with severely impaired kidney function. Sci Rep.

[323] Drucker D.J. (2016). The cardiovascular biology of glucagon-like peptide-1. Cell Metabol.

[324] Ghosal S., Myers B., Herman J.P. (2013). Role of central glucagon-like peptide-1 in stress regulation. Physiol Behav.

[325] Meguro S., Sano M., Kawai T. (2012). A new preventive strategy for hypoglycemia incorporating added food diet in patients with type 2 diabetes who received sitagliptin therapy. Endocr Res.

[326] Brown N.J. (2012). Cardiovascular effects of antidiabetic agents: focus on blood pressure effects of incretin-based therapies. J Am Soc Hypertens.

[327] Zhang X., Zhao Q. (2016). Effects of dipeptidyl peptidase-4 inhibitors on blood pressure in patients with type 2 diabetes: a systematic review and meta-analysis. J Hypertens.

[328] Ribeiro-Silva J.C., Marques V.B., Dos Santos L. (2023). Effects of dipeptidyl peptidase 4 inhibition on the endothelial control of the vascular tone. Am J Physiol Cell Physiol.

[329] Fioretto P., Zambon A., Rossato M., Busetto L., Vettor R. (2016). SGLT2 inhibitors and the diabetic kidney. Diabetes Care.

[330] Salvatore T., Galiero R., Caturano A. (2022). An overview of the cardiorenal protective mechanisms of SGLT2 inhibitors. Int J Mol Sci.

[331] Ashfaq A., Meineck M., Pautz A., Arioglu-Inan E., Weinmann-Menke J., Michel M.C. (2023). A systematic review on renal effects of SGLT2 inhibitors in rodent models of diabetic nephropathy. Pharmacol Ther.

[332] Kasina S.V.S.K., Baradhi K.M. (2025). Dipeptidyl peptidase IV (DPP IV) inhibitors. *StatPearls [Internet]*. https://www.ncbi.nlm.nih.gov/pubmed/31194471.

[333] Sayiner Z.A., Okyar B., Kısacık B., Akarsu E., Özkaya M., Araz M. (2018). DPP-4 inhibitors increase the incidence of arthritis/arthralgia but do not affect autoimmunity. Acta Endocrinol (Buchar).

[334] Yang W., Cai X., Zhang S., Han X., Ji L. (2021). DPP-4 inhibitor treatment and the risk of bullous pemphigoid: a systematic review and meta-analysis. Diabetes Metab Res Rev.

[335] U.S. Food and Drug Administration (2014). FDA adds warnings about heart failure risk to labels of type 2 diabetes medicines. Drug Safety Communications. https://www.fda.gov/drugs/drug-safety-and-availability/fda-drug-safety-communication-fda-adds-warnings-about-heart-failure-risk-labels-type-2-diabetes#:∼:text=%5B%204%2D5%2D2016%20%5D,have%20heart%20or%20kidney%20disease.

[336] Nauck M.A., Muus Ghorbani M.L., Kreiner E., Saevereid H.A., Buse J.B., Investigators LPCobotLT (2019). Effects of liraglutide compared with placebo on events of acute gallbladder or biliary disease in patients with type 2 diabetes at high risk for cardiovascular events in the LEADER randomized trial. Diabetes Care.

[337] Baxter S.M., Lund L.C., Andersen J.H. (2025). Glucagon-like peptide 1 receptor agonists and risk of thyroid cancer: an international multisite cohort study. Thyroid.

[338] U.S. Food and Drug Administration (2015). FDA revises labels of SGLT2 inhibitors to include warnings about ketoacidosis and UTIs. Drug Safety Communications. https://www.fda.gov/drugs/drug-safety-and-availability/fda-revises-labels-sglt2-inhibitors-diabetes-include-warnings-about-too-much-acid-blood-and-serious#:∼:text=%5B12%2D4%2D2015%5D,of%20serious%20urinary%20tract%20infections.

[339] U.S. Food and Drug Administration (2018). FDA warns about rare occurrences of Fournier’s gangrene with SGLT2 inhibitors. Drug Safety Communications. https://www.fda.gov/drugs/drug-safety-and-availability/fda-warns-about-rare-occurrences-serious-infection-genital-area-sglt2-inhibitors-diabetes.

[340] Bersoff-Matcha S.J., Chamberlain C., Cao C., Kortepeter C., Chong W.H. (2019). Fournier gangrene associated with sodium-glucose cotransporter-2 inhibitors: a review of spontaneous postmarketing cases. Ann Int Med.

[341] Krepostman N., Kramer H. (2021). Lower urinary tract symptoms should be queried when initiating sodium glucose cotransporter 2 inhibitors. Kidney360.

[342] Munzel T., Sinning C., Post F., Warnholtz A., Schulz E. (2008). Pathophysiology, diagnosis and prognostic implications of endothelial dysfunction. Ann Med.

[343] Schachinger V., Britten M.B., Zeiher A.M. (2000). Prognostic impact of coronary vasodilator dysfunction on adverse long-term outcome of coronary heart disease. Circulation.

[344] Griendling K.K., FitzGerald G.A. (2003). Oxidative stress and cardiovascular injury: part II: animal and human studies. Circulation.

[345] Griendling K.K., FitzGerald G.A. (2003). Oxidative stress and cardiovascular injury: part I: basic mechanisms and in vivo monitoring of ROS. Circulation.

[346] Munzel T., Gori T., Bruno R.M., Taddei S. (2010). Is oxidative stress a therapeutic target in cardiovascular disease?. Eur Heart J.

[347] Zhang L., Zalewski A., Liu Y. (2003). Diabetes-induced oxidative stress and low-grade inflammation in porcine coronary arteries. Circulation.

[348] Lonn M.E., Dennis J.M., Stocker R. (2012). Actions of “antioxidants” in the protection against atherosclerosis. Free Radic Biol Med.

[349] Mikkelsen R.R., Hundahl M.P., Torp C.K. (2022). Immunomodulatory and immunosuppressive therapies in cardiovascular disease and type 2 diabetes mellitus: a bedside-to-bench approach. Eur J Pharmacol.

[350] Das A.K., Kalra S., Tiwaskar M. (2019). Expert group consensus opinion: role of anti-inflammatory agents in the management of type-2 diabetes (T2D). J Assoc Physicians India.

[351] Aristizabal-Colorado D., Ocampo-Posada M., Rivera-Martinez W.A. (2024). SGLT2 inhibitors and how they work beyond the glucosuric effect. state of the art. Am J Cardiovasc Drugs.

